# Mechanical force application to the nucleus regulates nucleocytoplasmic transport

**DOI:** 10.1038/s41556-022-00927-7

**Published:** 2022-06-09

**Authors:** Ion Andreu, Ignasi Granero-Moya, Nimesh R. Chahare, Kessem Clein, Marc Molina Jordàn, Amy E. M. Beedle, Alberto Elosegui-Artola, Juan F. Abenza, Leone Rossetti, Xavier Trepat, Barak Raveh, Pere Roca-Cusachs

**Affiliations:** 1 Institute for Bioengineering of Catalonia (IBEC), the Barcelona Institute of Technology (BIST), 08028 Barcelona, Spain; 2 Universidad de Navarra, TECNUN Escuela de Ingeniería, Manuel de Lardizabal 15, 20018, Donostia-San Sebastián, Spain; 3 Universitat de Barcelona, 08036 Barcelona, Spain; 4 School of Computer Science and Engineering, The Hebrew University of Jerusalem, 9190416, Jerusalem, Israel; 5 Department of Physics, King’s College London, London, WC2R 2LS, UK; 6 Harvard John A. Paulson School of Engineering and Applied Sciences, Harvard University, Cambridge, MA, 02138, USA; 7 Cell and Tissue Mechanobiology Laboratory, The Francis Crick Institute, London, NW1 1AT UK; 8 Institució Catalana de Recerca i Estudis Avançats (ICREA), Passeig de Lluís Companys, 23, 08010 Barcelona; 9 Centro de Investigación Biomédica en Red en Bioingeniería, Biomateriales y Nanomedicina (CIBER-BBN), 08028 Barcelona, Spain

## Abstract

Mechanical force controls fundamental cellular processes in health and disease, and increasing evidence shows that the nucleus both experiences and senses applied forces. Such forces can lead to the nuclear translocation of proteins, but whether force controls nucleocytoplasmic transport, and how, remains unknown. Here we show that nuclear forces differentially control passive and facilitated nucleocytoplasmic transport, setting the rules for the mechanosensitivity of shuttling proteins. We demonstrate that nuclear force increases permeability across nuclear pore complexes, with a dependence on molecular weight that is stronger for passive than facilitated diffusion. Due to this differential effect, force leads to the translocation into or out of the nucleus of cargoes within a given range of molecular weight and affinity for nuclear transport receptors. Further, we show that the mechanosensitivity of several transcriptional regulators can be both explained by this mechanism, and engineered exogenously by introducing appropriate nuclear localization signals. Our work unveils a mechanism of mechanically induced signalling, likely operating in parallel to others, with potential applicability across signalling pathways.

## Introduction

Cells sense and respond to mechanical stimuli from their environment by a process known as mechanosensing, which drives important processes in health and disease^1–3^. Growing evidence shows that the cell nucleus is directly submitted to force^4–6^, and can act as a mechanosensor^7^. Force applied to the nucleus (henceforth termed nuclear force for simplicity) can affect chromatin architecture^8^, the accessibility of the transcription machinery^9^, the conformation of nucleoskeletal proteins such as lamins^10^, or cell contractility^11,12^. Further, forces transmitted to cells, and specifically nuclei, affect the nucleocytoplasmic localization of transcriptional regulators involved in different signalling pathways^13^. As proposed for MRTF-A^14,15^, β-catenin^16,17^, or YAP^18–20^, this can be due to a retention mechanism, in which force controls the localization of proteins by regulating their affinity for binding partners in the nucleus or cytoplasm. Alternatively, the nuclear translocation of YAP^21^ and MyoD^22^ has been associated to a force-induced increase in passive diffusion across nuclear pore complexes (NPCs). From this evidence, it is tempting to hypothesize that nucleocytoplasmic transport could be mechanosensitive *per se*, independently of any specific signalling pathway. This would enable a general mechanism by which nuclear force could control the nuclear localization of proteins, and thereby transcription. However, mere changes in passive diffusion can provide neither directionality nor molecular specificity, so whether there is such a mechanism, and how it operates, remains unknown.

Nucleocytoplasmic transport takes place through NPCs in two main ways, passive and facilitated diffusion^23,24^. Passive diffusion is rapid for small proteins, but is progressively impaired as the molecular weight (MW) of the protein increases^25–27^. This impairment is caused by a meshwork of disordered proteins within NPCs called phenylalanine-glycine (FG) Nups, commonly termed the NPC permeability barrier^28^. Facilitated diffusion of larger proteins is mediated by nuclear transport receptors (NTRs)^29,30^, which interact specifically with both the cargo molecules and FG Nups to overcome the NPC permeability barrier. They are divided between importins (mediating active nuclear import) and exportins (mediating active nuclear export)^31^. Both classes interact with cargoes by binding to specific sequences^32^ termed nuclear localisation signals (NLS) or nuclear export signals (NES) for proteins binding to importins or exportins, respectively^33,34^. The directionality of facilitated transport in either the import or export direction is enabled by the coupling of binding/unbinding events to the phosphorylation status of the small GTPase Ran (either GTP, predominant in the nucleus, or GDP, predominant in the cytoplasm)^30^. For example, in the canonical import, a complex is formed between importin β (which interacts with FG Nups), importin α (which binds importin β), and the cargo (which binds importin α through an NLS). The complex then diffuses through the NPC and finally dissociates in the nucleus in a RanGTP-dependent manner^31,32^.

## Results

### Nucleocytoplasmic transport is mechanosensitive

**Figure 1:**
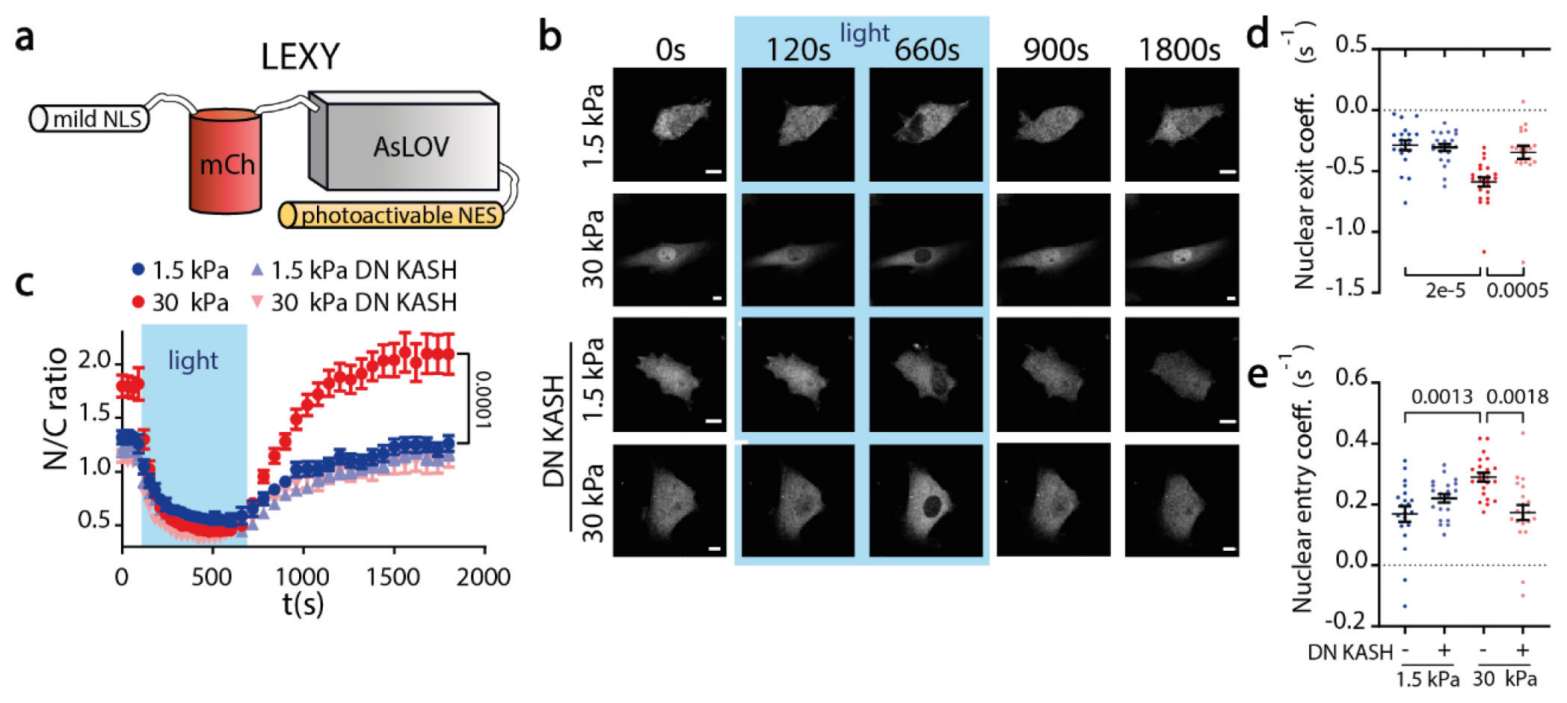
Nucleocytoplasmic transport is mechanosensitive. **a)** Cartoon of light-activated nucleocytoplasmic shuttling construct. Mild NLS is always active, NES is activated only upon light excitation. **b)** Time sequences of construct fluorescence before, during, and after excitation for cells seeded on 1.5/30 kPa substrates, with or without DN KASH overexpression. Scale bars, 20 μm. **c-e)** Corresponding quantifications of N/C ratios, and coefficients of exit and subsequent re-entry of constructs into the nucleus (in units of s^-1^, obtained by fitting an exponential to the curves, see methods). (N=20, 22, 21, 21 cells per condition (1.5 kPa, 30 kPa, 1.5 kPa DN KASH, and 30 kPa DN KASH, respectively) from 3 independent experiments, data are presented as mean values +/- SEM.In c) the bar indicates the statistical significance between the last timepoint of 1.5kPa and 30kPa values. In d-e, p-values calculated with 2-way ANOVA Šídák’s multiple comparisons test. Source numerical data are available in source data.

To assess if and how mechanical force affects nucleocytoplasmic transport, we studied different artificial constructs undergoing both passive and facilitated diffusion, transfected in mouse embryonic fibroblasts (MEFs). First, we used a light-inducible nuclear export construct (LEXY)^35^ (Fig. 1a). The construct presents a mild NLS fused to mCherry, plus a stronger NES that is only functional upon light excitation. To control the mechanical environment, cells were seeded on soft or stiff fibronectin-coated polyacrylamide gels (Young’s modulus of 1.5 and 30 kPa, respectively). Increasing substrate stiffness leads to the growth of focal adhesions, increasing the transmission of actomyosin-generated forces between cells and the substrate^36,37^. In turn, these forces reach and deform the nucleus through the Linker of Nucleus and Cytoskeleton (LINC) complex^21,38^, which connects actin fibres to the nuclear lamina. Before photoactivation (t=0), with only the NLS active, the nuclear to cytoplasmic ratio (N/C ratio) was higher for cells on stiff substrates (Fig. 1b,c). Upon excitation by light, the construct exited the nucleus to similar final N/C ratios in both conditions, although the rate of N/C change was higher for the stiff substrate (Fig. 1b-d). Once light excitation stopped, the reverse process occurred, with N/C ratios increasing faster for the stiff substrate, until restoring original values (Fig. 1e). We then co-transfected cells with DN-KASH, a dominant-negative domain of nesprin that disrupts the LINC complex^4^ and prevents force transmission to the nucleus^6^. DN-KASH overexpression led cells on stiff substrates to behave like those on soft substrates (Fig. 1b-e), demonstrating that the effect of stiffness was mediated by nuclear force.

### Passive diffusion is mechanosensitive for small MWs

**Extended data figure 1:**
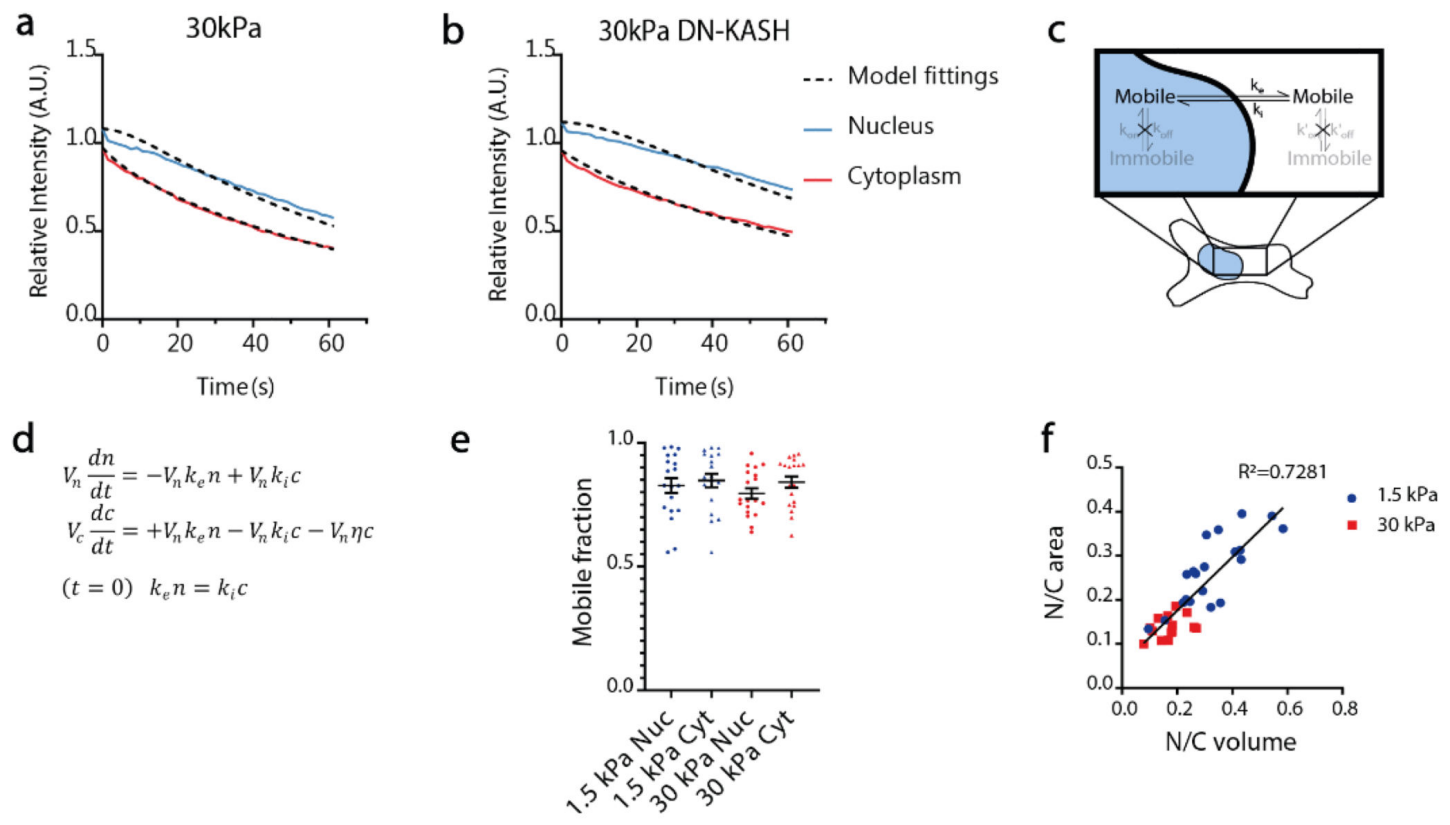
**a,b)** Examples of curves showing fluorescence intensity as a function of time in the nucleus and cytoplasm in FLIP experiments on two example cells transfected with the diffusive 41kDa construct and seeded on a) 30 kPa in control condition and b) 30kPa with DN-KASH overexpression. Data represent the mean fluorescence intensity of the compartments (nucleus/cytoplasm), normalized with the mean of the whole cell before the beginning of photobleaching, and corrected for background signal. Each curve depicts a representative experiment of one cell each. **c,d)** Cartoon and equations describing the model used for fitting curves as in A,B, and calculating influx and efflux rates. The model considers the molecules to freely diffuse inside the nuclear and cytoplasmic compartments (see methods). **e**) Mobile fraction of the L_NLS 41kDa construct in the nucleus (Nuc) and cytoplasm (Cyt) of cells seeded on 1.5/30 kPa gels. N=19 cells from 3 independent experiments, lines show mean ±SEM **f**) For cells seeded on 1.5 and 30 kPa gels, correlation between nuclear to cytosolic ratios of volume, and of areas as measured in confocal slices used for FLIP measurements; regression equation *y = 0,6075 x + 0,05375*. N=20 (1.5kPa) and N=14 (30kPa) cells from 2 independent experiments. Black line shows the linear regression. Source numerical data are available in source data.

**Figure 2:**
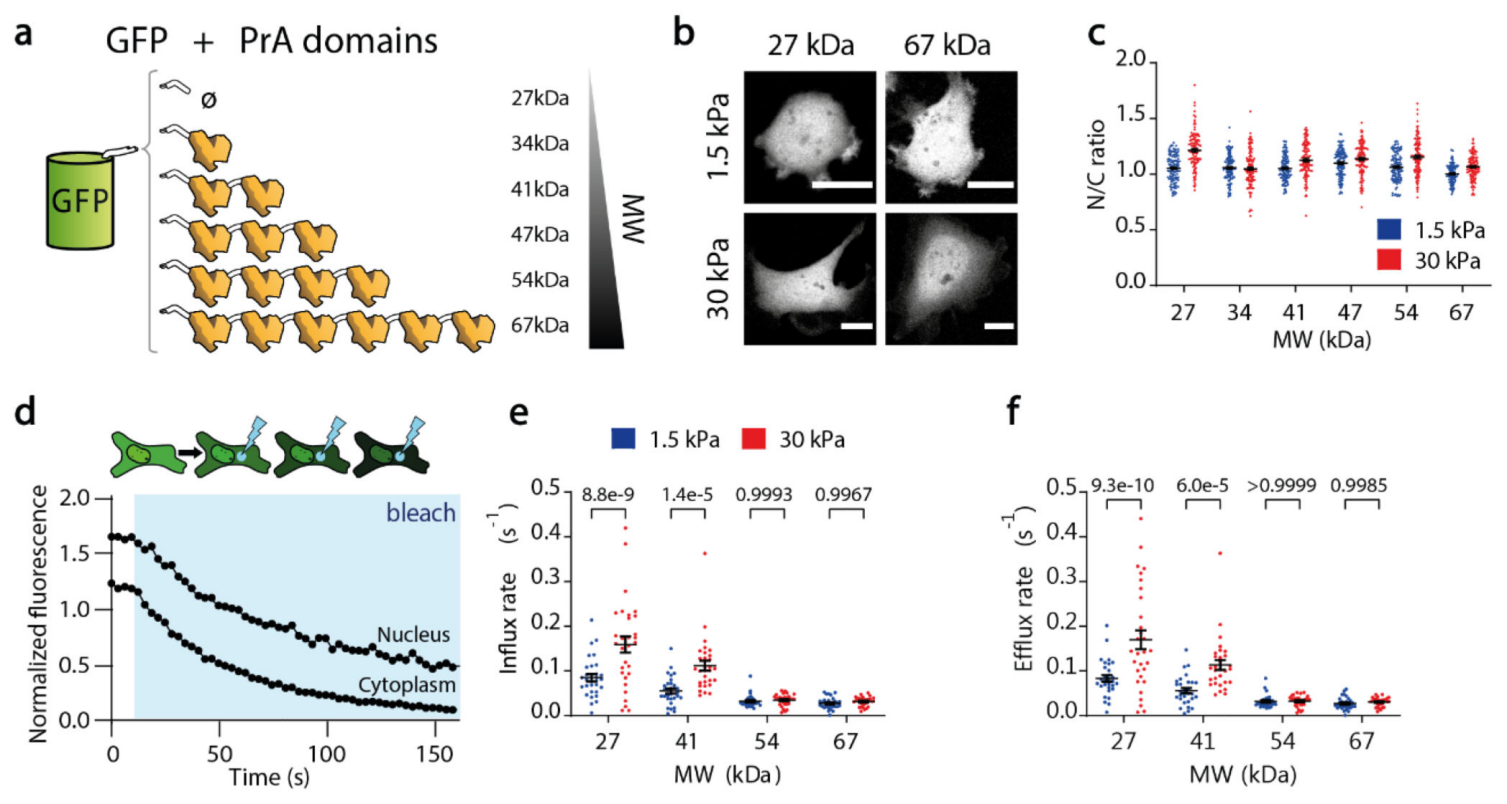
Passive diffusion through NPCs is mechanosensitive for small MWs. **a)** Cartoon of constructs with EGFP and different amount of repeats of PrA domains. **b)** Images showing fluorescence of indicated constructs on 1.5/30 kPa substrates. **c)** N/C ratios of constructs on 1.5/30 kPa substrates as a function of MW. N=120 cells from 3 independent experiments. Significant effects of stiffness and MW were observed (p <1e-15 and p <1e-15; computed via 2-way ANOVA). **d)** Example of a FLIP experiment: a laser photobleaches a region of the cell cytoplasm, and fluorescence intensities are recorded over time in nucleus and cytoplasm. Resulting curves are fitted to a kinetic model to obtain influx and efflux rates (see methods). **e,f)** Influx and efflux rates on 1.5 and 30 kPa substrates as a function of MW of the constructs. N=30 cells from 3 independent experiments. The effects of both substrate stiffness and MW were significant in both e,f). p-values e) 2.9e-8, <1e-15, f) 4.0e-8, <1e-15, computed via 2-way ANOVA. Scale bars, 20 μm. Data are mean ±SEM. Source numerical data are available in source data.

Our results strongly suggest that nucleocytoplasmic transport is generally affected by nuclear force, but do not clarify the contributions of passive and facilitated diffusion (the ~45 KDa LEXY construct is likely sufficiently small to diffuse passively). To dissect the different contributions, we first used constructs undergoing only passive diffusion, and regulated their diffusivity through their MW. These constructs were composed of a Green Fluorescent Protein (GFP), attached through a short linker to between zero and six repeats of the 7 kDa bacterial Protein A (PrA) (Fig. 2a). PrA is inert and purely diffusive in eukaryotic cells, as shown previously^26^ and also confirmed by the complete fluorescence recovery of the constructs after photobleaching (Extended Data Fig. 1e). When we transfected the constructs in cells, the N/C ratios of all proteins were ≈ 1 regardless of MW and substrate stiffness (Fig. 2 b,c).

**Extended data figure 2:**
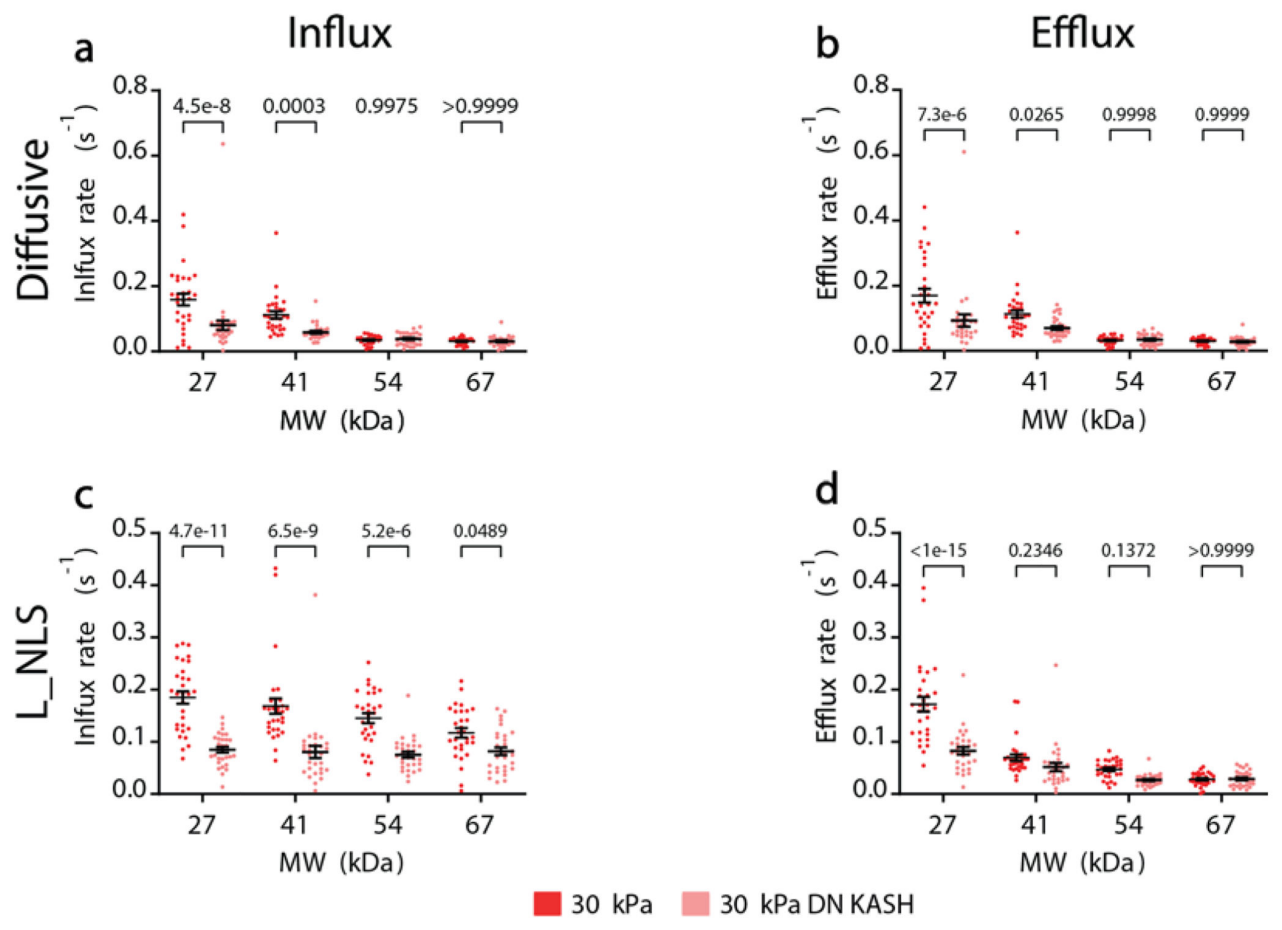
**a,b)** Influx and efflux rates of diffusive constructs for cells seeded on 30 kPa gels, with or without DN-KASH overexpression. In a, both MW (p<1e-15) and DN KASH (p=1e-6) effects tested significant. In b, both MW (p<1e-15) and DN KASH (p=0,0002) effects tested significant. **c,d)** Influx and efflux rates of constructs containing L_NLS for cells seeded on 30 kPa gels, with or without DN-KASH overexpression. In c, both MW (p=0,0025) and DN KASH (p<1e-15) effects tested significant. In d, both MW (p<1e-15) and DN KASH (p=3.4e-10) effects tested significant. In all panels, N= 30 cells from 3 independent experiments. Two-way ANOVA, Šídák’s multiple comparisons test was used to obtain p-values between conditions. Data are mean ±SEM. Source numerical data are available in source data.

This result shows that concentrations of passively diffusing proteins were not mechanosensitive (where mechanosensitivity is defined as the fold change in a given magnitude in stiff versus soft substrates). However, this does not provide information on diffusion kinetics. To quantify this, we adapted a previously described method and model^20^ based on Fluorescence Loss in Photobleaching (FLIP, Fig. 2d), which allowed us to measure nuclear influx and efflux rates (see methods and Extended Data Fig. 1). These rates quantify overall transport into and out of the nucleus, regardless of whether it is passive or mediated by active import/export. As expected, both influx and efflux rates decreased with MW (Fig. 2e,f). Interestingly, rates increased with substrate stiffness, and this effect decreased for increasing MW (Fig. 2e,f). Confirming that this was mediated by nuclear force, DN-KASH overexpression had the same effect as reducing substrate stiffness (Extended Data Fig. 2). Thus, nuclear force weakens the permeability barrier of NPCs (i.e., increases diffusion), and the effect is more pronounced for molecules with low MW (high diffusivity). Nevertheless, and because diffusion is non-directional, this does not affect the steady state nucleocytoplasmic distribution of molecules, which remains uniform.

### Mechanosensitivity of facilitated vs passive diffusion

**Figure 3:**
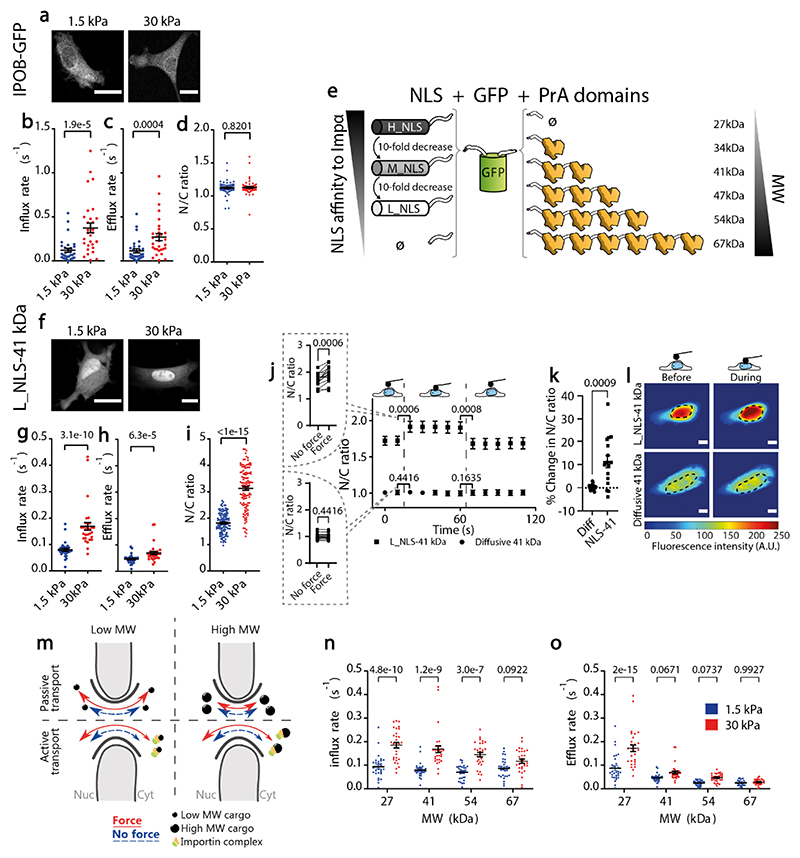
Differential mechanosensitivity of facilitated import versus passive diffusion explains force-induced nuclear translocation. **a)** Example importin β-GFP images for cells on 1.5/30 kPa substrates. **b-d)** Corresponding importin β-GFP influx rates (b), efflux rates (c), and resulting N/C ratios (d). N=30, 30, and 60 cells from 3 independent experiments. p-values calculated with two-tailed Mann-Whitney test. **e)** Cartoon of constructs with EGFP, different number of repeats of PrA domains, and NLS of different affinities to importin α. **f)** Example images of L_NLS-41 kDa construct for cells on 1.5 and 30 kPa substrates. **g-i)** Corresponding Influx rates (g), efflux rates (h), and resulting N/C ratios (i) of L_NLS-41 kDa construct. N=30, N=30, N=120 cells from 3 independent experiments respectively each. p-values calculated with two-tailed Mann-Whitney test. **j)** N/C ratios of L_NLS-41 kDa or diffusive 41 kDa constructs in cells seeded on 1.5 kPa gels before, during, and after nuclear deformation with AFM. Graphs on the left show paired dot plots of the time points right before and after force application. p-values were calculated with two-tailed paired t-test. **k**) Corresponding % change in N/C ratios right after force application for both constructs. p-value was calculated with a two-tailed unpaired t-test with Welch’s correction. In j,k, N= 16 cells from 3 independent experiments, traces of all cells are shown in Extended Data Fig. 8. **l**) Corresponding images of constructs before and during force application, dotted line marks nucleus outline. Scale bars 20μm. **m)** Cartoon summarizing the effects of nuclear force and MW on active and passive transport. Passive transport decreases with MW, and depends on force only for low MW molecules. Active transport does not depend on MW, and depends on force regardless of MW. Note that active transport arrows also show a small arrow in the export direction, as discussed in the text. **n)** Influx rates (mediated by facilitated transport) of L_NLS constructs with different molecular weights. The effect of substrate stiffness and MW tested p<1e-15 and p=0.0004. **o)** Efflux rates of L_NLS constructs (mediated by passive transport) with different molecular weights. The effect of substrate stiffness and MW tested p=3,5e-11 and p<1e-15. In n), o), N= 30 cells from 3 independent experiments. Two-way ANOVA, Šídák’s multiple comparisons test was used to obtain p-values between conditions. Data are mean ±SEM in all panels. Source numerical data are available in source data.

Next, we assessed how substrate stiffness affected facilitated transport. We first assessed the protein directly interacting with FG Nups, importin β. As expected, transfected importin β-GFP localized at the nuclear membrane (Fig. 3a). Due to this localization and the diffraction limit, our FLIP measurements could not capture the likely very fast kinetics taking place in the immediate vicinity of the nuclear membrane. However, we did measure the kinetics of importin β molecules released in the bulk of either the nucleus or cytoplasm. Influx and efflux rates of importin β showed a high mechanosensitivity (Fig. 3b,c), similarly to that of highly diffusive passive molecules (Fig. 2e,f). Because importin β exhibits facilitated diffusion both in the influx and efflux direction, influx and efflux rates were largely symmetrical, leading to uniform concentrations inside and outside the nucleus regardless of substrate stiffness (Fig. 3d).

Then, we studied cargo proteins undergoing facilitated diffusion by adding NLS sequences to the GFP-PrA constructs (Fig. 3e). To regulate facilitated diffusion, we used NLS sequences with point mutations resulting in varying levels of affinity for importin α^39^. We termed them H_NLS, M_NLS, and L_NLS, for high, medium, and low affinity, respectively (see Supplementary Table 2). The mechanosensitivity of such constructs can be predicted from the behaviour of passively diffusing molecules (Fig. 2e,f) and importin β (Fig. 3b,c). Indeed, a cargo molecule with an NLS should have a high mechanosensitivity in the influx direction (because it enters the nucleus with importin β), but a low mechanosensitivity in the efflux direction if its MW is above ~ 40 kDa (because it exits the nucleus through passive diffusion, which loses mechanosensitivity as MW increases).

**Extended data figure 3:**
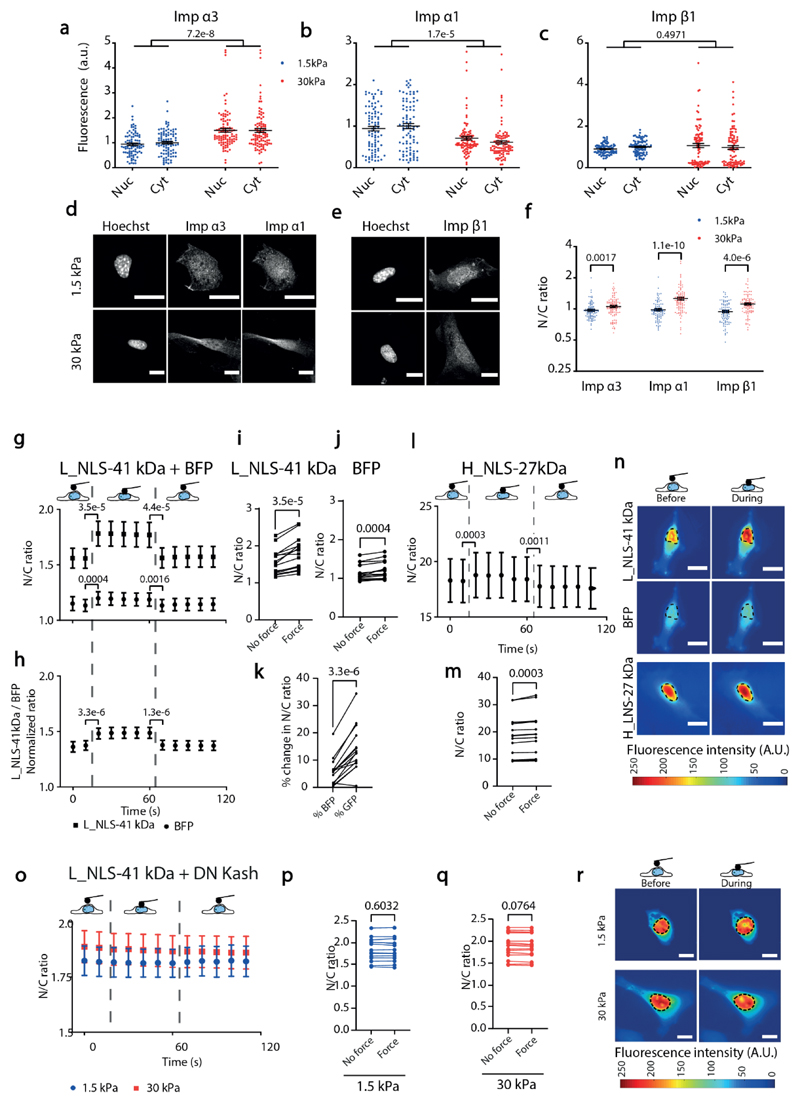
**a-c)** Average fluorescence intensities of nuclear and cytoplasmic areas of cells seeded on substrates of 1.5 or 30 kPa stiffness and immunostained for importin α3 (imp α3) importin α1 (imp α1), and importin β1 (imp β1). N= 90 cells from 3 independent experiments. The effect of substrate stiffness tested significant for importin α3 (p=7.2e-8) and importin α1 (p=1.7e-5), but not for importin β1 (p=0.4971). p-values from Two-way ANOVA **d-e)** Corresponding example images showing the nucleus (Hoechst) and the distribution of the different importins. **f)** Corresponding quantification of nuclear to cytoplasmic ratio of importin localization. N= 91,98, 91, 98, 90, 90 cells (from left to right) from 3 independent experiments. p-values from independent two-tailed Mann-Whitney tests. **g)** N/C ratios of L_NLS-41 kDa or BFP constructs in cells seeded on 1.5 kPa gels before, during, and after nuclear deformation with AFM. **h)** L_NLS-41 kDa ratios normalized by BFP ratios, from panel g) paired measures. **i,j)** from g, corresponding paired dot plots of the time points right before and after force application. **k)** from g, corresponding % change in N/C ratios right after force application for both constructs. In g,h,i,j,k N= 15 cells from 3 independent experiments, p-values were calculated with a two-tailed paired t-test. **l)** N/C ratios of H_NLS-27 kDa construct in cells seeded on 1.5 kPa gels before, during, and after nuclear deformation with AFM. **m)** from l, corresponding paired dot plots of the time points right before and after force application. In l, m, N= 15 cells from 3 independent experiments. p-values were calculated with a two-tailed paired t-test. **n)** Corresponding images of constructs before and during force application, dotted line marks nucleus outline. **o)** N/C ratios of the L_NLS-41 kDa construct in cells co-transfected with DN-KASH and seeded on 1.5 or 30 kPa gels before, during, and after nuclear deformation with AFM. Data are mean ±SEM. **p,q)**from o, corresponding paired dot plots of the time points right before and after force application. In o,p,q, N= 15 cells from 3 independent experiments. p-values were calculated with a two-tailed paired t-test, traces of all cells are shown in Extended Data Fig. 8. **r)** Corresponding images of constructs before and during force application, dotted line marks nucleus outline. Scale bars, 20 μm. Note: in AFM experiments, non-mechanosensitive constructs (BFP and H_NLS) still show a small increase with force, likely due to lensing effects caused by changes in cell shape during indentation. This increase (~6% for BFP, ~2% for H_NLS) is much smaller than that of the mechanosensitive construct (L_NLS 41 kDa, ~14%), see panel k. Panel h in fact shows the response of the L_NLS construct after factoring out the response of BFP. Data are mean ±SEM in all panels. Source numerical data are available in source data.

By taking L_NLS-EGFP-2PrA (41 kDa) as a starting point, we confirmed this prediction: this molecule had a higher mechanosensitivity in influx than efflux rates, leading to an increase in N/C ratios with stiffness (Fig. 3f-i). We then carried out several controls to confirm that this was caused by nuclear force. First, we checked that the same effects on rates were observed when comparing cells with and without DN-KASH overexpression (Extended Data Fig. 2). Second, we assessed stiffness-mediated changes in importin concentrations. Concentrations of importin β did not change with stiffness, but the two types of importin α binding to our NLS constructs (importin α3 and importin α1) respectively showed a ~50% increase or ~40% decrease with stiffness (Extended Data Fig. 3a-e). The N/C ratios of all importins remained close to 1 in all cases, with only a ~10-30% increase with stiffness that if anything should impair, rather than promote, nuclear import of cargo (Extended Data Fig. 3). Thus, changes in importin concentration may have an impact, but do not exhibit any consistent trend that could explain our results. Finally, we applied force to the nucleus of cells seeded on soft gels with an Atomic Force Microscope (AFM), and verified that this also led to an increase in N/C ratios only if the construct contained the L_NLS sequence (Fig. 3j-l). Applying force to cells co-transfected with L_NLS-EGFP-2PrA and purely diffusive BFP also led to a nuclear enrichment of GFP versus BFP (Extended Data Fig. 3). Response to AFM-applied force was also lost for cells overexpressing DN-KASH, showing that the effects of force require an intact LINC complex (Extended Data Fig. 3).

For L_NLS-EGFP-2PrA, nuclear accumulation with force is explained by a higher mechanosensitivity of facilitated versus passive diffusion. This differential behaviour may arise from the role of MW. Indeed, passive diffusion is strongly impaired as MW increases^26^ whereas facilitated diffusion can transport large molecules^40–42^. Thus, one could expect a scheme (summarized in Fig. 3m) in which passive diffusion decreases both in magnitude and in mechanosensitivity as MW increases (as measured in Fig. 2e,f) whereas facilitated transport is not affected (or only mildly affected) by MW. To verify this hypothesis, we measured influx and efflux rates of constructs containing the L_NLS sequence and different MW (Fig. 3n,o). Indeed, influx rates (dominated by active transport, Fig. 3m) had a much milder dependence on MW than efflux rates (dominated by diffusion and with very similar behaviour to that of purely diffusive constructs, Fig. 3o).

### Molecular properties defining mechanosensitivity

**Figure 4:**
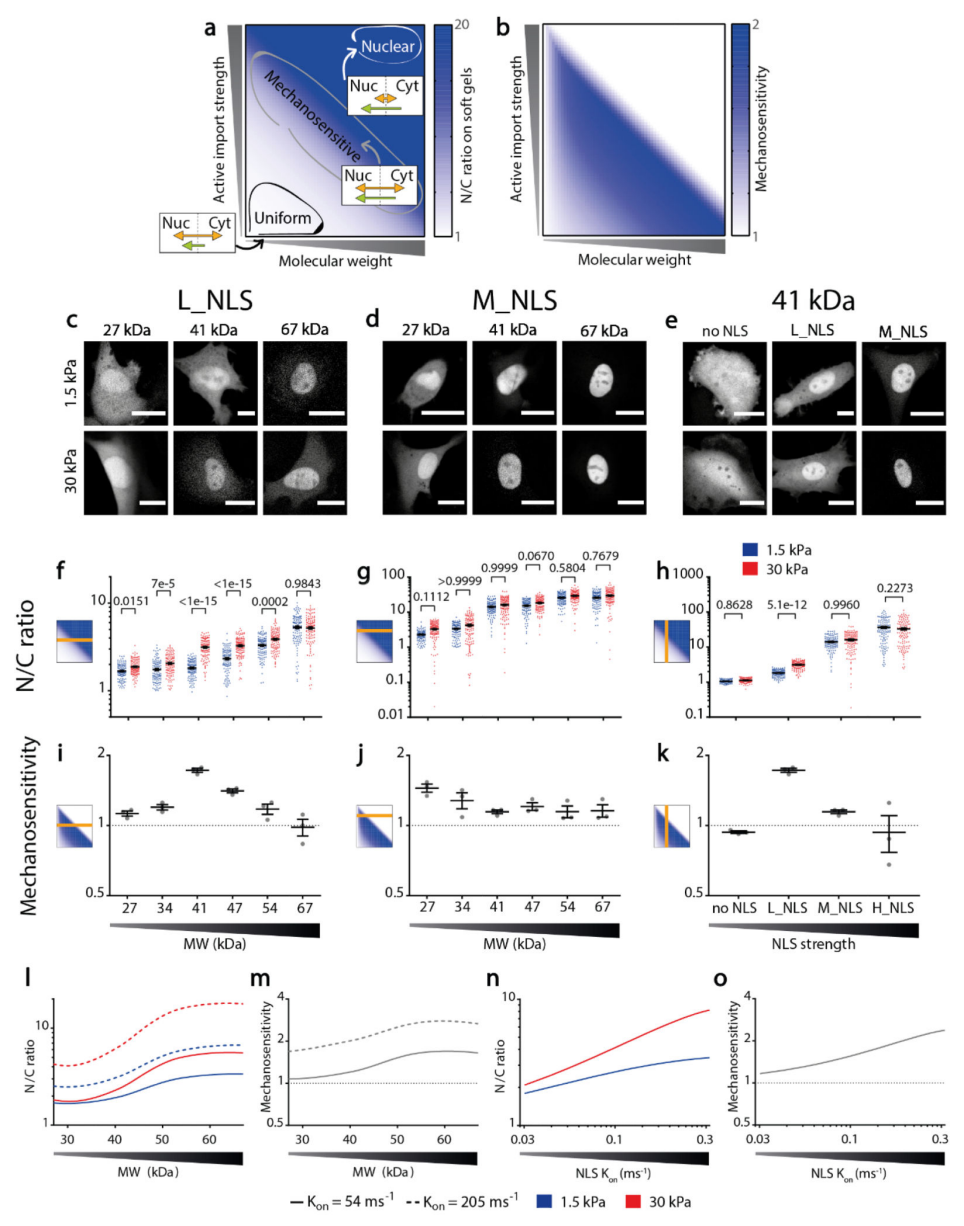
Balance between affinity to importins and MW defines the mechanosensitivity of nuclear localization. **a,b)** Qualitative prediction from conceptual model of how MW and affinity to importins should affect N/C ratios (a) on soft substrates and their mechanosensitivity (b) (see methods). Mechanosensitivity is defined as (N/C)_stiff_/(N/C)_soft_. **c-e)** Representative examples of construct distribution in cells seeded in substrates of 1.5kPa or 30kPa, for L_NLS constructs at different MW, M_NLS constructs at different MW, and 41kDa constructs at different NLS strengths. **f-h)** N/C ratios corresponding to the same conditions as C-E. **i-k)** Mechanosensitivity corresponding to the same conditions as C-E. **l-m)** Kinetic model predictions of N/C ratios (l) and mechanosensitivities (m) for NLS of different affinities for importin α (modelled through the binding rates k_on_ between the NLS and importin α, with values of 54 and 205 ms^–1^) as a function of MW. **n-o)** Model predictions of N/C ratios (n) and mechanosensitivities (o) for 41kDa constructs, as a function of increasing NLS strength. Statistics: f) N= 120 cells from 3 independent experiments. Both MW (p<1e-15) and Stiffness (p<1e-15) effects tested significant. g) N= 120 cells from 3 independent experiments. Both MW (p<1e-15) and Stiffness (p=0,0015) effects tested significant. h) N= 120 cells from 3 independent experiments. Both NLS strength (p<1e-15) and Stiffness (p=0,0012) effects tested significant. Two-way ANOVA, Šídák’s multiple comparisons test was used to obtain p-values between conditions. Scale bars: 20 μm. Data are mean ±SEM. Source numerical data are available in source data.

With these elements, we can generate an initial conceptual model of how nucleocytoplasmic transport should broadly depend on force, MW, and NLS affinity (see Supplementary Note). To this end, we assume that N/C ratios are given by the ratio of influx and efflux rates, where efflux rates are purely passive and influx rates have additive contributions of both passive and facilitated diffusion. Then, we assume as experimentally verified that **i)** passive influx and efflux rates (which are equal) decrease as MW increases, **ii)** passive influx and efflux rates increase when nuclear force is applied, but this effect disappears as MW increases, **iii)** facilitated influx rates increase with nuclear force and with NLS sequence affinity, but do not depend on MW. We also assume that there is a limit to the efficiency of active facilitated transport, and therefore **iv)** N/C ratios saturate and cannot increase above a given level. In such a saturation regime, changes in influx and efflux rates can no longer behave differently and should be matched. The potential origin of this is discussed in the more detailed, kinetic model introduced later in the manuscript. With these assumptions, we can plot two simple diagrams showing how N/C ratios should depend on MW and NLS affinity before applying force to the nucleus (Fig. 4a), and their fold change with force, i.e., their mechanosensitivity (Fig. 4b). According to this framework, for low MW or a weak NLS, passive diffusion dominates over facilitated import, leading to N/C ratios close to 1 independently of nuclear force. For high MW or a strong NLS, facilitated import dominates over diffusion, leading to high, saturated N/C ratios, also independently of nuclear force. However, when passive and facilitated rates are comparable they depend differently on force, leading to mechanosensitive N/C ratios. As MW decreases (and passive diffusion increases) a progressively higher facilitated influx is required to match passive diffusion, and thus the “mechanosensitive zone” is placed along a diagonal in Fig. 4b.

**Extended data figure 4:**
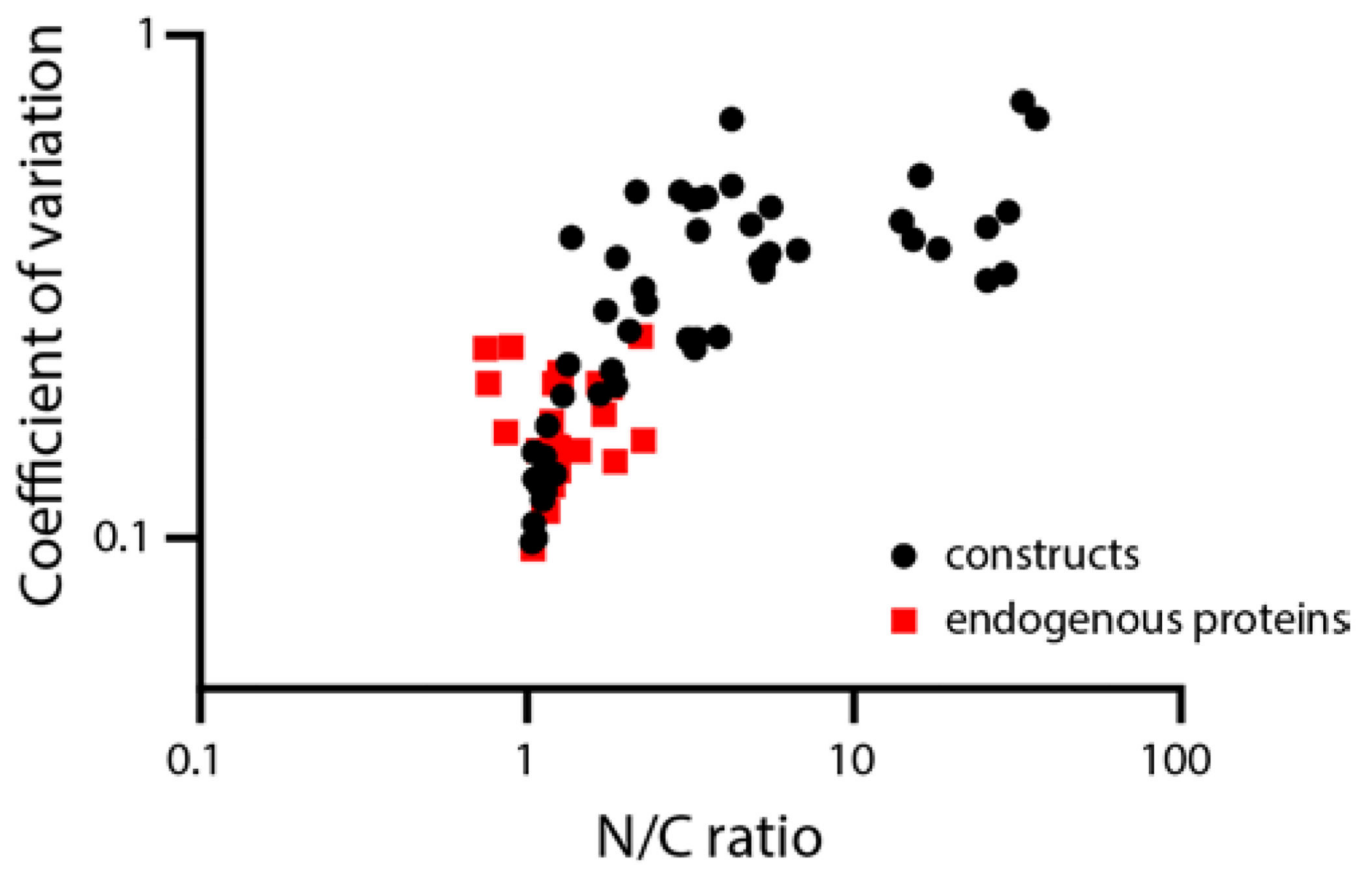
Relationship between mean N/C ratio as reported in figures, and corresponding coefficient of variation (standard deviation divided by the mean). The different points show all different constructs and conditions reported in the manuscript. Black dots indicate values of overexpressed engineered constructs, red squares indicate values of stained endogenous proteins. Source numerical data are available in source data.

We then verified the different predictions of the conceptual model by using the different constructs. First, for proteins with a fixed NLS sequence (L_NLS), N/C ratios increased with MW monotonically, but mechanosensitivity peaked at an intermediate MW between the high passive diffusion regime (low MW) and the saturated regime (high MW) (Fig. 4c,f,i). Of note, increasing N/C ratios also led to increased variability in measurements, due to the increased noise caused by the low cytoplasmic signal (Extended Data Fig. 4). Second, increasing MW in proteins with a fixed NLS sequence of higher affinity (M_NLS) moved the point of maximum mechanosensitivity to a lower MW (Fig. 4d,g,j). Finally, increasing NLS affinity in proteins with a fixed MW (41 kDa) also increased N/C ratios monotonically, but affected mechanosensitivity in a biphasic manner (Fig. 4e,h,k). For this last set of constructs, we also used the highly nuclear and not mechanosensitive H_NLS construct to verify that force application with AFM did not lead to the same response as in mechanosensitive constructs (Extended Data Fig. 3).

Our conceptual model thus provides a useful framework to interpret our results, but it does not consider important elements of nucleocytoplasmic transport, such as the Ran cycle, or the fact that facilitated transport is reversible and can operate in both directions^43^. To address this, we developed a more elaborate kinetic mathematical model, which follows a canonical description of importin-mediated nucleocytoplasmic transport. This includes docking, undocking, and bidirectional translocation of importins in different intermediate forms, competitive binding of cargo and RanGTP to importins, the Ran cycle, and passive diffusion of unbound cargo molecules (see Supplementary Note)^31,44–46^. To model the effect of force on passive diffusion, we used the experimentally measured passive diffusion rates as a function of force and MW from fig. 1e,f. For facilitated diffusion, we simply assumed that force reduces the mean time required for importin-cargo complexes to cross NPCs (in a MW-independent way), without changing any other parameter.

**Extended data figure 5:**
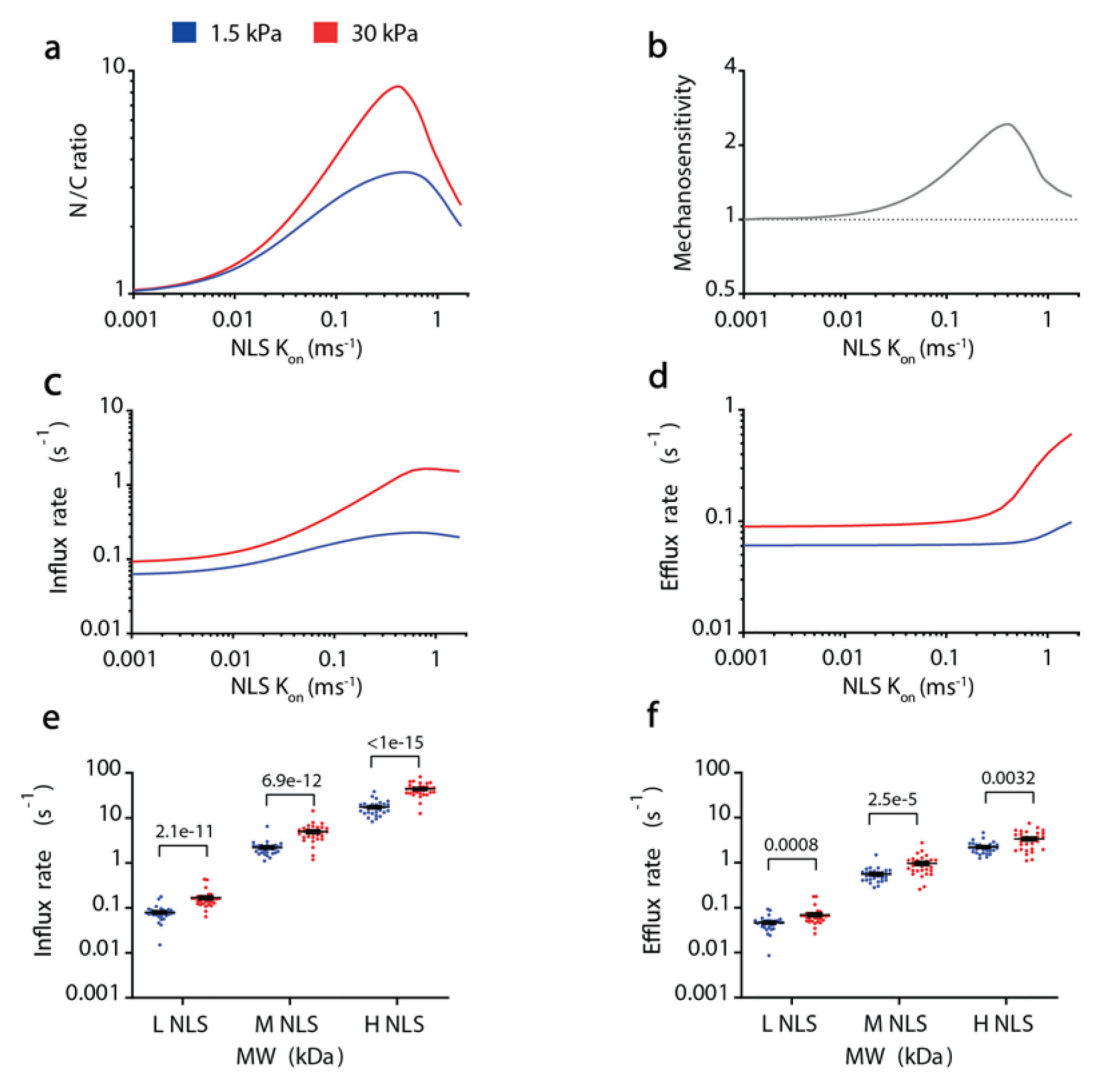
**(a-d)** Model predictions for N/C ratios (a), mechanosensitivities (b), influx rates (c) and efflux rates (d) for 41kDa constructs as a function of NLS affinity (modelled by the binding rate *k_on_* between the NLS and importin α). **e-f)** Experimental Influx and efflux rates of 41 kDa constructs containing NLS signals of different affinity for importin β. In both cases (e,f), NLS strength and substrate stiffness effects tested significant (respectively: e) p<1e-15, p<1e-15, f) p<1e-15, p=2.4e-10). N= 30 cells from 3 independent experiments. p-values from Two-way ANOVA. Data are mean ±SEM. Source numerical data are available in source data.

The kinetic model correctly predicted the increase of N/C ratios, and of their mechanosensitivity, with MW and NLS affinity (Fig. 4l-o). Interestingly, as NLS affinities increase, the model predicted an increase not only in influx rates but to a lesser degree also efflux rates, something which we confirmed experimentally (Extended Data Fig. 5). This occurs because as NLS affinity increases, cargo molecules can compete with Ran-GTP for binding importins, limiting the ability of Ran-GTP to disassemble the cargo-importin complex. This leads to the facilitated diffusion of importin-cargo complexes out of (and not only into) the nucleus. Eventually and for very high NLS affinities, the model predicted that N/C ratios would first saturate and then collapse, as cargo becomes so tightly bound to importins that it diffuses with it out of the nucleus regardless of Ran-GTP (Extended Data Fig. 5). This was not observed in experiments, and likely corresponds to non-physiological high affinities. The only experimental feature that the kinetic model did not capture was the fact that high MWs or NLS affinities decreased mechanosensitivity (Fig. 4i-k). Instead, the model predicted that mechanosensitivity should be maintained even in this regime (Fig. 4m,o). Potentially, this could be because the model underestimated the effect of NLS affinity on efflux rates (Extended Data Fig. 5). If efflux rates are mediated by facilitated rather than passive diffusion, then their dependency on force is the same as that of influx rates, and the overall effect on N/C ratios cancels out.

### Mechanosensitivity of facilitated export

**Extended data figure 6:**
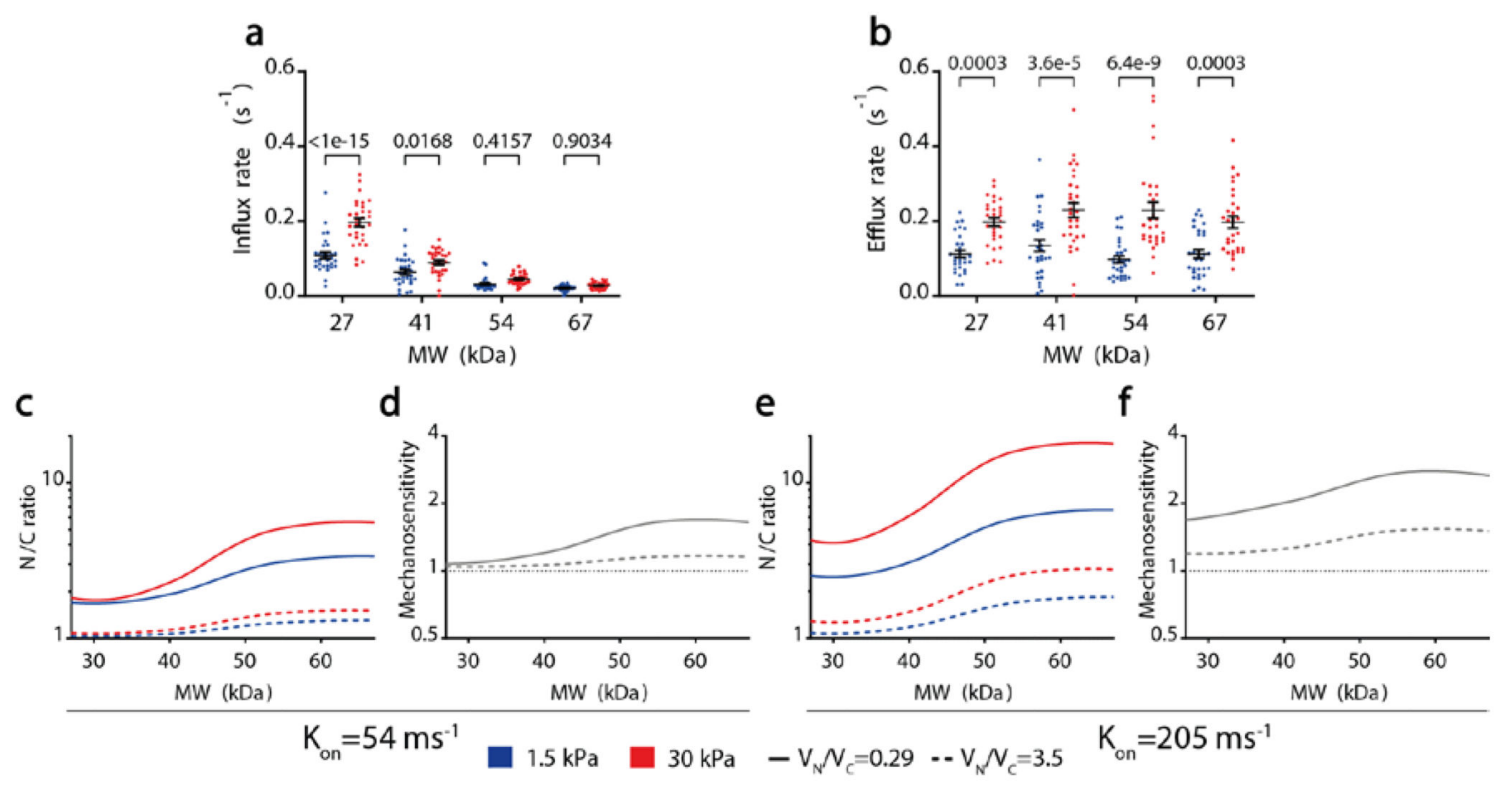
For M_NES constructs, influx rates (mediated by passive transport) and efflux rates (mediated by facilitated transport) as a function of molecular weight. N= 30 cells from 3 independent experiments. Substrate stiffness effects tested significative in both cases (a) p=5.1e-13; b) p<1e-15); MW only tested significative for influx, a) p<1e-15; b) p=0.2138). Two-way ANOVA, Šídák’s multiple comparisons test was used to obtain p-values between conditions. Data presented as mean ±SEM. **c-d)** Model predictions of N/C ratios (c) and mechanosensitivities (d) for an NLS with a binding rate k_on_ of 54 ms^-1^ as a function of MW. Data are shown for experimentally measured N/C volume ratios (0.29) and for inverted volume ratios (3.5). **e-f)** Same predictions as in c,d for an NLS with a binding rate kon of 205 ms^-1^. Note that these predictions simply evaluate the role of N/C volumes on import, they do not explicitly model the export cycle (and hence mechanosensitivities are above and not below 1). Source numerical data are available in source data.

**Figure 5:**
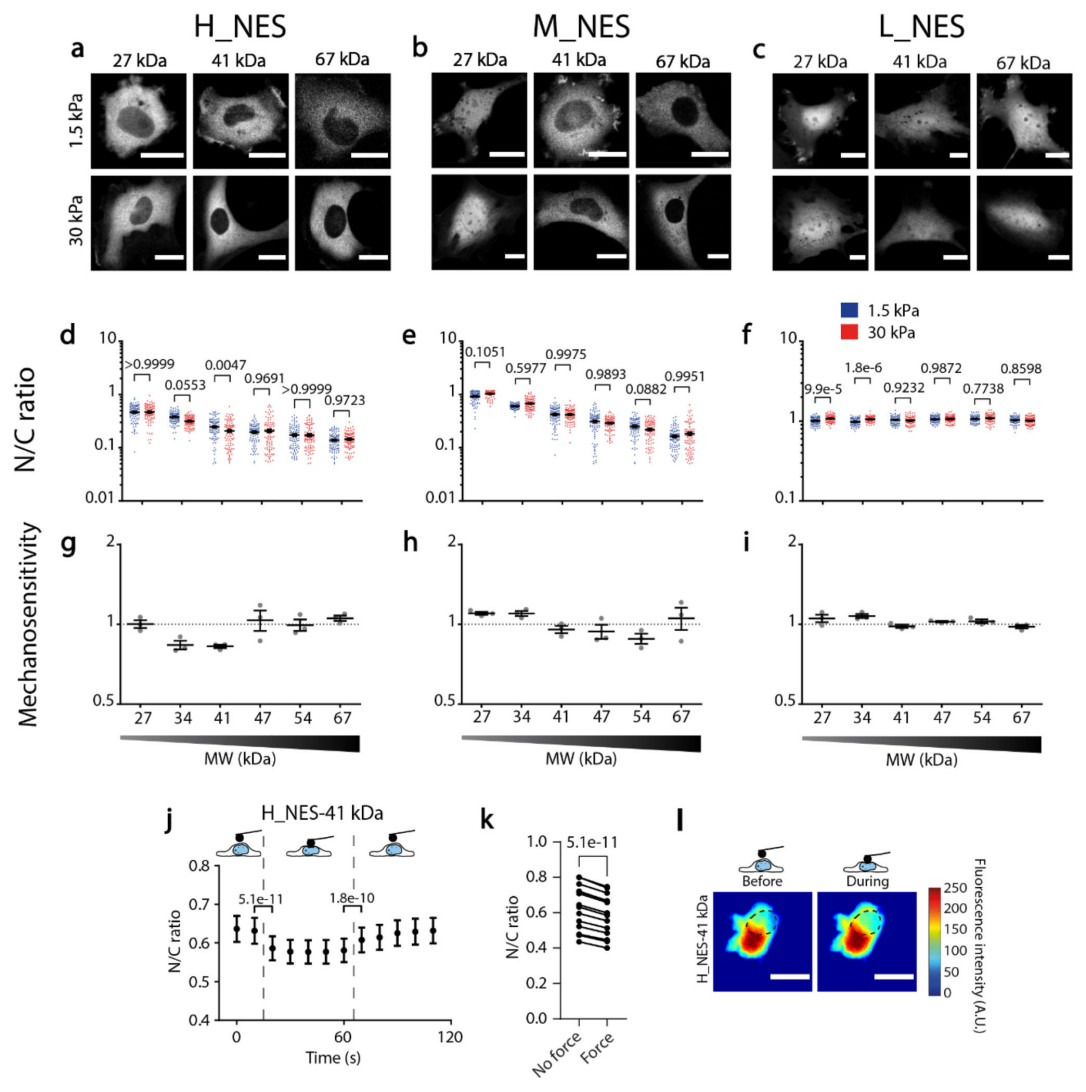
Balance between affinity to Exportin1 and MW defines the mechanosensitivity of nuclear localization in constructs containing NES signals. **a-c)** Representative examples of construct distribution in cells seeded in substrates of 1.5kPa or 30kPa, for H_NES constructs at different MW, M_NES constructs at different MW, and L_NES constructs at different MW. **d-f)** N/C ratios corresponding to the same conditions as A-C. d) N= 90 cells from 3 independent experiments. Both MW (p<1e-15) and Stiffness (p=0,0162) effects tested significant. e) N= 120 cells from 3 independent experiments. Only MW effects tested significant (p<1e-15). f) N= 90 cells from 3 independent experiments. Both MW (p<1e-15) and Stiffness (p=0,0001) effects tested significant. Two-way ANOVA, Šídák’s multiple comparisons test was used to obtain p-values between conditions. **g-i)** Mechanosensitivity corresponding to the same conditions as A-C. Mechanosensitivity is defined as (N/C)_stiff_/(N/C)_soft_ (n=3 experiments)**. j)** N/C ratios of H_NES 41 kDa construct in cells seeded on 1.5 kPa gels before, during, and after nuclear deformation with AFM. **k)** From data in j, paired dot plots of the time points right before and after force application. In j and k, N= 15 cells from 3 independent experiments. p-values were calculated with a two-tailed paired t-test, traces of all cells are shown in Extended Data Fig. 8. **l)** Corresponding images of constructs before and during force application, dotted line marks nucleus outline. Scale bars 20μm. Data are mean ±SEM. Source numerical data are available in source data.

Given the observed mechanosensitivity of active nuclear import, one might expect a similar (but reversed) behaviour for active export. To test this, we developed constructs by combining PrA repeats with different NES signals of different strength^47^ (see Supplementary Table 2). N/C ratios changed as expected with MW and NES strength (by following the opposite trends than NLS constructs, Fig. 5a-i). The mechanosensitivity of the constructs also behaved in the opposite way, with constructs leaving (rather than entering) the nucleus with force (Fig. 5g-i). Consistently, influx and efflux rates of NES constructs also had opposite trends with MW than NLS constructs: efflux rates were largely independent of MW, whereas influx rates showed a strong dependence, mimicking diffusive constructs (Extended Data Fig. 5a,b). Confirming the effect of force, applying force to the nucleus with AFM to the most mechanosensitive NES construct (H_NES-EGFP-2PrA 41 KDa) led to a reduction of N/C ratios (Fig. 5j-l). Interestingly, mechanosensitivity of the NES constructs was systematically milder than that of the NLS constructs. This is consistent with the behaviour of the light inducible construct (Fig. 1b), which had a stiffness-dependent localization when controlled by active import (no light excitation) but not when controlled by active export (under light excitation). This lower mechanosensitivity of active export as compared to import may be related to the many differences between the transport cycles in both directions, and particularly the fact that NES-mediated export, unlike NLS-mediated import, is directly coupled to the hydrolysis of Ran-GTP^31,44,48^. However, another potential intuitive explanation could be that a concentration gradient is more easily generated by accumulating proteins in a small compartment (the nucleus) than a large one (the cytoplasm). In line with this hypothesis, model predictions obtained by inverting nuclear and cytoplasmic volumes led to lower N/C ratios and mechanosensitivity (Extended Data Fig. 6).

### Mechanosensitivity of transcriptional regulators

**Extended data figure 7:**
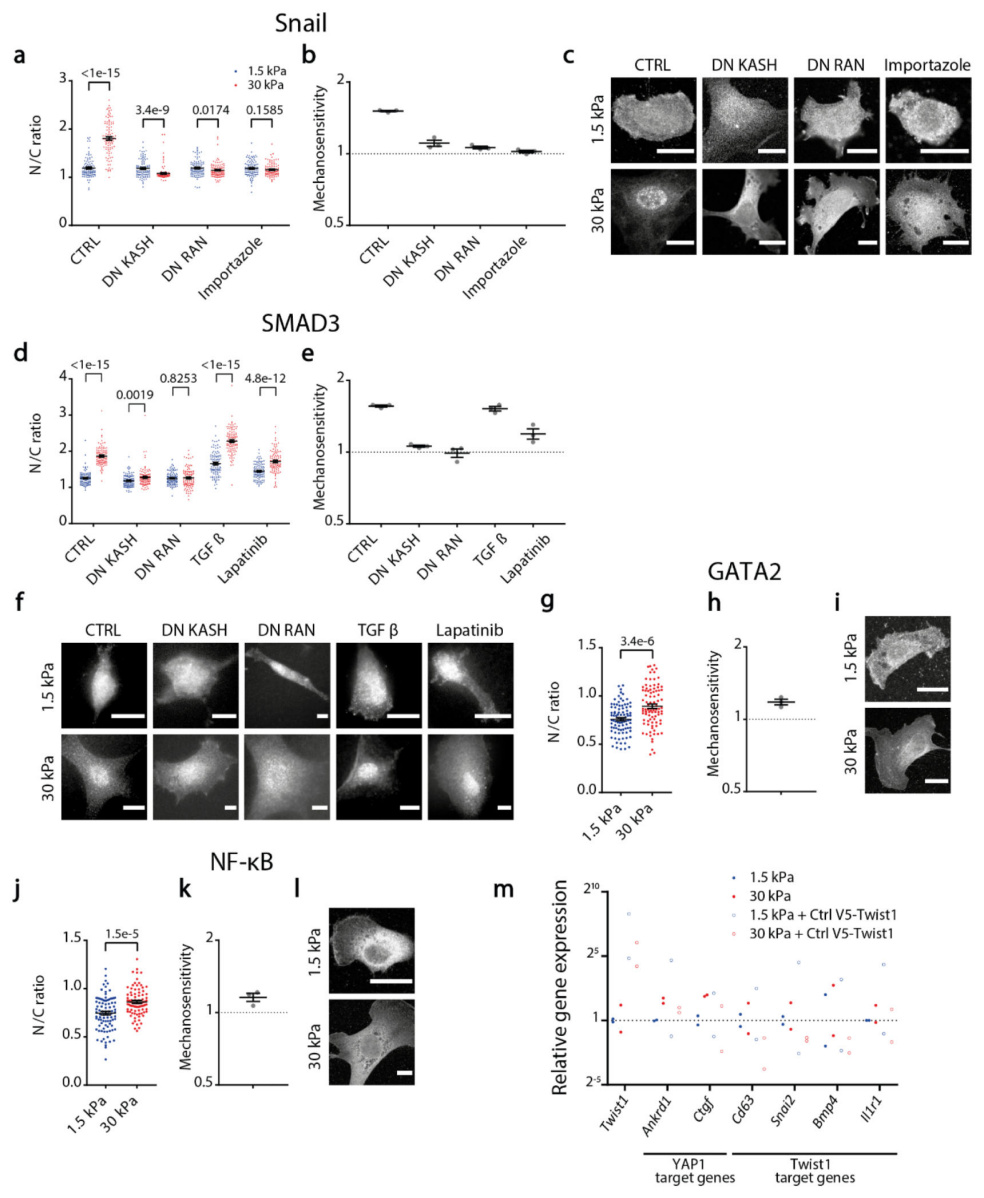
**a-c)** For Snail stainings at different conditions, quantifications of N/C ratios on 1.5/30 kPa substrates (a, N= 100 cells from 3 independent repeats), corresponding mechanosensitivities for the 3 different repeats (b), and representative images (c). **d-f)** For SMAD3 stainings at different conditions, quantifications of N/C ratios on 1.5/30 kPa substrates (d, N= 100 cells from 3 different repeats), corresponding mechanosensitivities for the 3 different repeats (e), and representative images (f). **g-i)** For GATA2 stainings at different conditions, quantifications of N/C ratios on 1.5/30 kPa substrates (g, N= 90 cells from 3 independent repeats), Corresponding mechanosensitivities for the 3 different repeats (h), and representative images (i). **j-l)** For NF-κβ stainings at different conditions, quantifications of N/C ratios on 1.5/30 kPa substrates, (j, N= 90 cells from 3 independent repeats), corresponding mechanosensitivities for the 3 different repeats (k), and representative images (l). For a-l, data are presented as mean ±SEM, scale bars correspond to 20 μm, and p-values from corrected multiple two-tailed Mann-Whitney (a,d) and two-tailed Mann-Whitney (g,j) tests. **m)** Relative gene expression of different genes as assessed with qPCR. Conditions are cells seeded on 1.5 or 30 kPa substrates, overexpressing or not a WT twist1 construct (Ctrl V5-twist1). Gene expression is shown relative to the 1.5 kPa condition without overexpression. n=2 independent experimental repeats. Source numerical data are available in source data.

**Figure 6:**
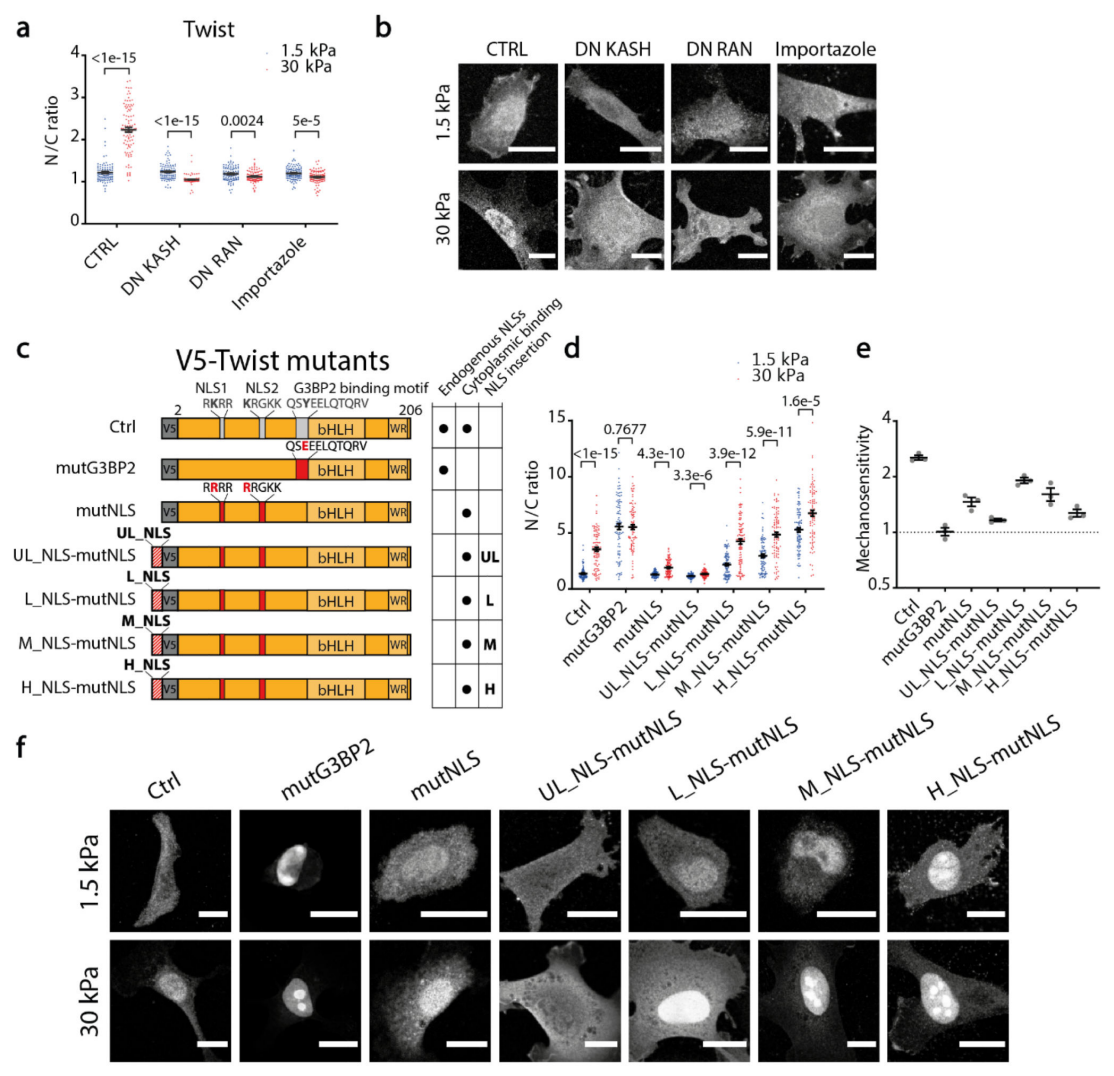
The mechanosensitivity of twist1 can be re-engineered with exogenous NLS sequences. **a)** N/C ratios of endogenous twist1 for cells on 1.5/30 kPa substrates, and under indicated treatments. N= 100 cells from 3 independent experiments. p-values from two-tailed Mann-Whitney tests, corrected for multiple tests in the intracondition comparisons with the two-stage step-up method of Benjamini, Krieger and Yekuteili. **b)** Corresponding images of twist1 distribution. **c)** Scheme of different twist1 mutants. Mutations inactivating both NLS sequences and the G3BP2 binding motif are indicated in red. **d)** N/C ratios of transfected twist1 mutants for cells on 1.5/30 kPa substrates. N= 90 cells from 3 independent experiments. p-values from two-tailed Mann-Whitney tests, corrected for multiple tests with the two-stage step-up method of Benjamini, Krieger and Yekuteili. **e)** Corresponding construct mechanosensitivities, defined as (N/C)_stiff_/(N/C)_soft_ (N= 3 experiments). **f)** Corresponding images showing the distribution of the different mutants. Scale bars, 20 μm, data are mean ±SEM. Source numerical data are available in source data.

Finally, we evaluated whether nucleocytoplasmic transport can explain the reported mechanosensitivity of different transcriptional regulators. Different transcriptional regulators localize to the nucleus with force in different contexts, including YAP^6,49^, twist1^50^, snail^51^, SMAD3^52^, GATA2^53^, and NFκβ^54^. If their mechanosensitivity is explained by regulation of nucleocytoplasmic transport with nuclear force, then it should be abolished by preventing either force transmission to the nucleus (by overexpressing DN-KASH) or nucleocytoplasmic transport (by overexpressing either DN-Ran, a dominant-negative version of Ran^55^, or by treatment with importazole, a drug which blocks active import by importin β^56^). For the case of YAP, we previously showed that its mechanosensitivity is abrogated by both factors^6^. Regarding the rest, GATA2 and NFκβ exhibited a very low mechanosensitivity in our system (Extended Data Fig. 7), but SMAD3, Snail, and Twist1 showed a clear response (Extended Data Fig. 7 and Fig. 6a,b). In all cases, mechanosensitivity was abrogated by DN-KASH, DN-Ran, or importazole (Extended Data Fig. 7 and Fig. 6a,b). Interestingly and consistent with our finding that NLS constructs were more mechanosensitive than NES constructs, SMAD3 mechanosensitivity was higher for cells treated with TGFβ (which induces SMAD3 nuclear import) than with lapatinib (which induces SMAD3 nuclear export)^57^.

Thus, the mechanosensitivity of several transcriptional regulators is controlled by force-induced effects in nucleocytoplasmic transport. Our proposed mechanism also has the stronger implication that mechanosensitivity can be engineered simply by selecting the appropriate levels of affinity to importins. To verify this, we took twist1 as a convenient model, since its NLS sequences are known, and their function can be abolished with point mutations^58^. Further, its mechanosensitivity depends on its binding to G3BP2, which retains twist1 in the cytoplasm^50^. We first overexpressed wild-type twist1 in cells, which retained the mechanosensitivity of endogenous twist1 (Fig. 6c-f). Of note, changes in twist1 caused by either stiffness or overexpression did not consistently increase the expression of twist1 target genes (Extended Data Fig. 7). Thus, twist1 serves as a model for protein localization but not transcription. Then, we overexpressed a G3BP2 binding deficient mutant, mutG3BP2. As expected, this led to high N/C ratios on both soft and stiff substrates, thereby losing mechanosensitivity. Confirming the role of nucleocytoplasmic transport, the NLS dead mutant (mutNLS, still under the control of G3BP2), lost the nuclear localization in both soft and stiff substrates, thereby also losing mechanosensitivity (although not completely, Fig. 6c-f). We then assessed whether we could restore twist1 mechanosensitivity by rescuing twist mutNLS not with its endogenous NLS, but by exogenously adding our different characterized NLS sequences (plus an additional ultra-low affinity sequence, UL_NLS). Adding NLS sequences of different strength mimicked the effects seen in Fig. 4: as the NLS strength increased, nuclear localization progressively increased, and mechanosensitivity was highest at a low strength (L_NLS), where it was almost as high as in the endogenous case. Thus, simply substituting the endogenous twist1 NLS with an exogenous one of the appropriate strength, not regulated by any twist-1 related signalling mechanism, recapitulates its mechanosensitivity.

## Discussion

Our work shows that force regulates nucleocytoplasmic transport by weakening the permeability barrier of NPCs, affecting both passive and facilitated diffusion. Because MW affects more passive than facilitated diffusion, this generates a differential effect on both types of transport that enables force-induced nuclear (or cytosolic) localization of cargo. The mechanical weakening of the permeability barrier is most likely the consequence of NPC deformation, and we previously reported increased apparent NPC diameters for cells on stiff versus soft substrates^6^. Further, recent structural evidence has confirmed the deformability of NPCs. In NPCs, the meshwork of FG Nup proteins that conforms the permeability barrier is supported by the NPC inner ring, which is formed by 8 symmetric spokes^59,60^. Spokes have limited interactions with each other through flexible linker proteins^61^. This allows NPCs to dilate or constrict by changing the distance between spokes, as proposed a decade ago^62^ and as verified very recently^63,64^. Such dilation and constriction indeed occur in response to energy depletion or to changes in osmotic pressure, likely in response to changes in nuclear membrane tension^63^. This proposed direct regulation of NPC permeability with force is strongly supported by the immediate response observed in AFM experiments, the effects observed in passive diffusion, and the dependency on MW. On top of this mechanism, indirect effects mediated for instance by changes in importin α levels (Extended Data fig. 3) or by competition between cargoes for importin binding (as recently demonstrated between YAP and importin 7^65^) may play a role in different contexts.

Three important open questions emerge from our findings. First, how mechanical deformation of NPCs weakens the permeability barrier of FG Nups in both passive and facilitated diffusion, remains to be understood. The LINC complex may play an important role, as suggested by the fact that responses to stiffness (in which cells apply force to the nucleus through the cytoskeleton and the LINC complex) are larger than responses to more unspecific force application with an AFM. This is further supported by the abrogation of AFM responses upon DN-KASH overexpression. Second, the exact set of properties that confer mechanosensitivity to transcriptional regulators or other proteins remains to be fully explored. The different transcriptional regulators discussed here range in size from over 20 kDa (for twist) to over 60 kDa (for YAP), thereby encompassing almost the full range of weights analyzed with our designed constructs. However, diffusivity through NPCs depends not only on MW but also on surface charges^66^ and protein mechanical properties^67^, which could play major roles. Finally, why facilitated export is less affected than facilitated import may be related to the different volumes of nucleus and cytoplasm (as suggested by modelling in Extended Data Fig. 6), to the different interactions between importins and exportins with FG-nups^68^ or to the asymmetric manner in which NPCs deform^69^.

Our work demonstrates a general mechanism of mechanosensitivity, with incorporated specificity through molecular properties such as the NLS sequence and MW. Although other mechanisms (such as differential binding to nuclear or cytosolic proteins) can generate mechanosensitive nuclear translocation^70,71^, our mechanism is consistent with the behaviour of several transcriptional regulators, and has potential general applicability. Our findings suggest that interfering with nucleocytoplasmic transport may be an avenue to regulate or abrogate mechanically-induced transcription in several pathological conditions. Perhaps even more excitingly, they open the door to design artificial mechanosensitive transcription factors, to enable mechanical control of transcriptional programs at will.

## Methods

### Cell culture and reagents

Mouse embryonic fibroblasts (MEFs) were cultured as previously described^72^, using Dulbecco’s modified eagle medium (DMEM, Thermofischer Scientific, 41965-039) supplemented with 10% v/v FBS (Thermofischer Scientific, 10270-106), 1% v/v penicillin-streptomycin (Thermofischer Scientific, 10378-016), and 1.5% v/v HEPES 1M (Sigma Aldrich, H0887). Cell cultures were routinely checked for mycoplasma. CO_2_-independent media was prepared by using CO_2_-independent DMEM (Thermofischer Scientific, 18045054) supplemented with 10% v/v FBS, 1% v/v penicillin-streptomycin, 1.5% v/v HEPES 1M, and 2% v/v L-Glutamine (Thermofischer Scientific, 25030-024). Media for AFM experiments was supplemented with Rutin (ThermoFischer Scientific, 132391000) 10 mg/l right before the experiment. Importazole (Sigma Aldrich) was used at 40 μM concentration for 1 h^56^. Cells were transfected the day before the experiment using Neon transfection device (ThermoFischer Scientific) according to manufacturer’s instructions. Cells were seeded ~4 h before the experiment.

### Antibodies and compounds

For primary antibodies, we used Anti Twist antibody (Twist2C1A, Santa cruz, sc-81417, RRID:AB_1130910) 1:200, Mouse monoclonal antibody to SNAIL + SLUG - N-terminal (clone number: CL3700; abcam, ab224731) 1:200, rabbit polyclonal anti SMAD3 (Cell Signaling, 9513, RRID:AB_2286450) 1:40, Rabbit polyclonal antibody to GATA2 (Abcam, ab153820) 1:200, rabbit polyclonal Anti-NF-kB p65 antibody (abcam, ab16502, RRID:AB_443394) 1:200, KPNA4 / Importin alpha 3 (NBP1-31260 Novus Biologicals, RRID:AB_2133841) 1:200, KPNA2 / Importin alpha 1 (MAB6207 Bio-techne, Clone number: 682239) 1:200, KPNB1 / Importin Beta 1 (ab2811 Abcam, RRID:AB_2133989) 1:200. The secondary antibodies used were Alexa Fluor 488 anti-mouse (A-11029; Thermo Fischer Scientific, RRID:AB_2534088) and Alexa Fluor 555 anti-rabbit (A-21429; Thermo Fischer Scientific, RRID:AB_2535850) diluted 1:200.**Plasmids**

If not specified otherwise, plasmids were constructed via standard molecular biology methods. **LEXY plasmids:** NLS-mCherry-LEXY (pDN122) was a gift from Barbara Di Ventura & Roland Eils (Addgene plasmid # 72655 ; http://n2t.net/addgene:72655 ; RRID:Addgene_72655)^35^. **Nuclear transport plasmids:** NLS, NES, or nought combinations with different molecular weight modules were designed as following: Localization signal plus GGGGS linker, EGFP, and different repeats of Protein A (PrA) from *Staphylococcus aureus* modules. Nuclear Localization Signal sequences were extracted from Hodel *et al*.(2001)^39^. Nuclear Export Signal sequences were extracted from Kanwal *et al*. (2004)^47^. Protein A domain sequences were used originally in Timney *et al*. (2016) ^26^ and were kindly provided by M. Rout. NLS and NES insertions were performed following Liu and Naisith protocol^73^. PrA insertions plasmid were constructed via Gibson Assembly protocol, as well as BFP plasmid from IG062. For more detailed information see tables S1 and S2. **DN-KASH DN-RAN:** DN (Dominant Negative)-KASH was described previously as EGFP-Nesprin1-KASH in Zhang *et al*., (2001)^74^. DN (Dominant Negative)-RAN (Addgene plasmid # 30309, described as pmCherry-C1-RanQ69L) was a gift from Jay Brenman^75^. **Twist mutants:** pBABE-puro-mTwist was a gift from Bob Weinberg (Addgene plasmid # 1783 ; http://n2t.net/addgene:1783 ; RRID:Addgene_1783)^76^. mTwist was cloned into a pEGFP-C3 backbone and a V5 tag was included at the N-terminal. The different mutants were constructed by adding the corresponding NLS sequences and/or changing the indicated codons. For more detailed information see Supplementary Table 1.

### Polyacrylamyde gels

Polyacrylamide gels were prepared as previously described^77^, and coated using a protocol adapted from the literature^78^. Gels were prepared by mixing acrylamide (5.5% or 12% v/v for 1.5 or 30 kPa gels, respectively) and Bis-acrylamide (0.04% or 0.15% v/v for 1.5 or 30 kPa gels, respectively) with 2% v/v 200-nm-diameter dark red fluorescence carboxylate-modified beads (Fluospheres, ThermoFischer Scientific), 0.5% v/v ammonium persulphate (APS, Sigma Aldrich), and 0.05% tetramethylethylenediamine (TEMED, Sigma Aldrich), in PBS 1X. A drop of 22 μl was placed on top of a glass bottom well and then sandwiched with an 18 mm diameter coverslip. Gels where then let for 45 min at room temperature to polymerize. Finally, gels were covered in PBS 1X and the top coverslip was removed. To coat gels, we first prepared a mixture containing HEPES (0.5M, pH 6, 10% v/v), Acrylamide and Bis-Acrylamide (BioRad), N-hydroxysuccinimide (NHS, 0.3% v/v from an initial solution of 10 mg/ml in dimethyl sulfoxide, Sigma Aldrich), Irgacure 2959 (1% v/v, BASF), and Di(trimethylolpropane)tetra-acrylate (0.0012% v/v, Sigma Aldrich), in milliQ water. This mixture was placed on top of gels, and gels were then illuminated with UV light for 10 minutes. After exposure, gels were washed once with HEPES 25mM Ph 6 and once with PBS. Gels were then incubated with 10 μg/ml of fibronectin in PBS overnight at 4ºC, UV treated in the hood for 10 minutes, washed once with PBS and immediately used. The rigidity (Young’s modulus) of the gels was measured as previously described^79^ using a Nanowizard 4 AFM (JPK). Silicon nitride pyramidal tips with an effective half angle θ of 20º and a nominal spring constant of k=0.01 N/m were used (MLCT, Bruker). The spring constant of the cantilevers was calibrated by thermal tuning using the simple harmonic oscillator model. Force-displacement curves with a peak-to-peak amplitude of 6 μm and a frequency of 1 Hz were acquired. 64 points near the gel centre were selected in each gel, separated 5 μm from each other. Eight gels produced in two batches were measured for each stiffness. To compute the Young’s modulus (E), the Hertz model equation for pyramidal tips was fitted to the force-displacement curves, using the JPK software (JPK Data Processing Version 6.1.79). The equation was fitted for an effective indentation of 500 nm.

### Immunostaining

Immunostainings were performed as previously described^6^. Cells were fixed with 4% v/v paraformaldehyde for 10 minutes, permeabilized with 0.1% v/v Triton X-100 for 40 minutes, blocked with 2% v/v Fish-Gelatin in PBS 1X for 40 minutes, incubated with primary antibody for 1 hour, washed 3 times with Fish-Gelatin-PBS for 5 minutes, incubated with secondary antibody for 1 hour, washed with Fish-Gelatin-PBS 3X for 5 minutes, and mounted using ProLong Gold Antifade Mountant (ThermoFischer Scientific).

### Real-time PCR experiments

Real-time PCR experiments were performed according to the manufacturer’s instructions (Applied Biosystems). Total mRNA was extracted from cells in the different conditions using the Qiagen RNeasy Micro Kit. Concentration of the obtained mRNA was measured with a Nanodrop ND-1000 Spectrophotometer. Equal amounts of RNA samples were reverse-transcribed into cDNA using the iScript^™^ cDNA Synthesis Kit. SYBR Green (Applied Biosystems 4385612) RT-qPCRs were performed in triplicates with a StepOnePlus System (Applied Biosystems) under standard conditions. The 2^–ΔΔC*t*^ method was used to calculate relative gene expression. All ΔΔC*t* values were normalized to the housekeeping gene *GAPDH*. Primer sequences for the different measured genes are detailed in Supplementary Table 3.

### Steady state image acquisition and analysis

Cells were imaged with a Nikon Eclipse Ti inverted confocal microscope with Micromanager (version 1.4.22), using a 60x water immersion objective 1.2 NA. Microscopy images were acquired by using Zeiss ZEN2.3 SP1 FP3 (black, version 14.0.24.201) or Micromanager (version 1.4.22). Nuclear to cytoplasmic (N/C) ratios were quantified manually by segmenting the nucleus using Hoechst (immunostaining) or taking advantage of the GFP tagged construct (live cells) by the following formula:

$$\frac{N}{C}=\frac{I_{nucleus}-I_{background\;}}{I_{cytoplasm\;}-I_{background\;}}$$

Where *I_nucleus_* and *I_cytoplasm_* are the mean fluorescence intensity of the nucleus and the cytoplasm respectively. ROIs in the nucleus an in the cytoplasm were selected manually next to each other, close to the nuclear membrane. *I_background_* is the mean intensity of the background far from the cell.

Mechanosensitivity was calculated once for each of the experimental repeats using the following formula:

$$mechanosensitivity=\frac{\left[N/C\;stiff\;substrate\right]}{\left[N/C\;soft\;substrate\right]}$$

Where *[N/C stiff substrate]* and *[N/C stiff substrate]* are the average N/C ratios on stiff/soft substrates for all cells within the experimental repeat. These quantifications were done by using ImageJ software (version 1.53e).

### Live cell AFM experiments

Live cell AFM experiments were carried out as previously described^6^. AFM experiments were carried out in a Nanowizard 4 AFM (JPK) mounted on top of a Nikon Ti Eclipse microscope, using the JPK software (JPK Data Processing Version 6.1.79). Polystyrene beads of 20 μm were attached using a non-fluorescent adhesive (NOA63, Norland Products) to the end of tipless MLCT cantilevers (Veeco). The spring constant of the cantilevers was calibrated by thermal tuning using the simple harmonic oscillator model. Experiments were carried out on cells previously transfected with the different constructs indicated in figures, incubated with Hoechst 33342 (Invitrogen), and seeded on 1.5 kPa gels. For each cell, the nucleus was identified by using the Hoechst fluorescence signal, and a force of 1.5 nN was applied to the nucleus. Once the maximum force was reached, the indentation was kept constant under force control, adjusting the z height by feedback control. An image was acquired every 10s by an Orca ER camera (Hamamatsu) and a 60X (NA = 1.2) objective.

### Photoactivation experiment and quantification

Photoactivation experiments were done with a Zeiss LSM880 inverted confocal microscope using a 63X 1.46 NA oil immersion objective and using using Zeiss ZEN2.3 SP1 FP3 (black, version 14.0.24.201). An argon laser was used with 561 nm wavelength for acquisition and 488 nm laser for stimulation. For experiments, 4 images were obtained before stimulation, followed by 19 images during stimulation, and 18 images during recovery. All images were acquired every 30 s. During the stimulation period, the 488 nm laser was irradiated to the whole field of view also every 30 s, during 1 s at 100% laser power.

To obtain the entry and exit coefficient a single exponential equation was fitted to the N/C ratio of each cell:

$$n/c(t)=(n/c{)}_{0}e^{-kt}$$

Where (*n/c*)_0_ is the initial ratio of the stimulation or recovery phase, *t* is time, and *k* is the entry or exit coefficient. The curve was fitted to the whole stimulation or recovery phase.

### FRAP Data Acquisition and Analysis

Estimation of mobile fraction of proteins was done using fluorescence recovery after photobleaching (FRAP) experiments. FRAP involves bleaching a region of interest (ROI) and then tracing the recovery of fluorescence in that region with respect to time. Image acquisition was done with a Zeiss LSM880 inverted confocal microscope objective and using using Zeiss ZEN2.3 SP1 FP3 (black, version 14.0.24.201), using a 63X 1.46 NA oil immersion objective and a 488nm wavelength argon laser at 100% laser power. We acquired images every 60 ms, before and after bleaching. We use two regions of interest (ROIs) for our experiments: first, the circular 14-pixel diameter (~6.9 μm^2^) region being bleached (ROIF). Second, the cell area segmented manually (ROIC). The data for ROIs consist of the fluorescence integrated density as a function of time from images acquired before and after photobleaching. For further analysis, we normalize the fluorescence intensities of ROIs using the double normalization method^80^. Double normalization corrects for photobleaching during the post bleach imaging, and normalizes recovery fluorescence with a pre bleach signal. Double normalized intensity (I) for recovery signal can be calculated by using following formula.

$$I=\frac{F}{F_{0}}\times \frac{C_{0}}{C}$$

where *F* and *C* are the fluorescence integrated densities of ROIF and ROIC respectively for post bleach imaging, and *F_0_* and *C_0_* correspond to pre bleach imaging. The mobile fraction *mf* represents the fraction of molecules that are free to diffuse. It is estimated by using the first timepoint after bleaching (*I_0_*) and the median of the last twenty timepoints (*I_f_*) in the following expression:

$$mf=\frac{I_{f}-I_{0}}{1-I_{0}}$$

### FLIP Model

Fluorescence loss in photobleaching (FLIP) is used to assess influx and efflux rates of the different constructs. FLIP experiments involve continually bleaching of a region of interest (ROIb) and tracking signal loss from different regions. Quantification of these curves yields the transport dynamics between nucleus and cytoplasm. We set up experiments and analysis motivated from^20^ for determining the rates of nuclear influx and efflux.

To model the FLIP data, we developed a system of Ordinary Differential Equations (ODEs) describing the change in protein concentration between two compartments i.e., the nucleus and the cytoplasm. These two compartments are linked with boundary fluxes going in (*Q_i_*) and out *(Q_e_)* of the nucleus (Fig S1).

We assume that the proteins remain in unbound and mobile state in each compartment. During steady state cells maintain a constant ratio (α) of protein concentration between nucleus (*n*) and cytoplasm (*c*), and the flux between both compartments is equal.

$$\begin{array}{l} \alpha =\frac{n}{c}\\ Q_{e}=Q_{i} \end{array}$$

During photobleaching the transport equations for the number of unbleached molecules in nucleus (*N*) and cytoplasm (*C*) can be described as follows, where *(Q_b_)* is the number of molecules being bleached per unit time.

$$\begin{array}{l} \frac{dN}{dt}=-Q_{e}+Q_{i}\\ \frac{dC}{dt}=+Q_{e}-Q_{i}-Q_{b} \end{array}$$

The fluxes are proportional to the concentration of the compartment, times a rate coefficient. Here, *k_e_*’, *k_i_*’ are efflux and influx rate coefficients respectively and η’ is the bleaching rate:

$$Q_{e}=k_{e}{\;}^{\prime }n\;Q_{i}=k_{i}{\;}^{\prime }c\;Q_{b}=\eta^{\prime }c$$

Because these rates (in units of volume per unit time) will depend on the size of the compartment, we define normalized rates as $k_{e}=k_{e}^{\prime }/V_{n},k_{i}=k_{i}^{\prime }/V_{n},\eta =\eta^{\prime }/V_{n}$, where *V_n_* is the volume of the nucleus. Note that we normalize both *k_e_* and *k_i_* by the same volume (that of the nucleus, *V_n_*) so that the values remain comparable, and that equal *k_e_* and *k_i_* correspond to equal concentrations in nucleus and cytoplasm. Thus:

$$Q_{e}=V_{n}k_{e}n\;Q_{i}=V_{n}k_{i}c\;Q_{b}=V_{n}\eta c$$

This enables us to rewrite transport equations in terms of concentration.

During bleaching,

$$\begin{array}{l} V_{n}\frac{dn}{dt}=-V_{n}k_{e}n+V_{n}k_{i}c\\ V_{c}\frac{dc}{dt}=+V_{n}k_{e}n-V_{n}k_{i}c-V_{n}\eta c \end{array}$$

Where *V_c_* is cytoplasm volume. During steady state,

$$\begin{array}{l} V_{n}k_{e}n=V_{n}k_{i}c\\ k_{e}\frac{n}{c}=k_{i}\\ k_{e}=\frac{k_{i}}{\alpha } \end{array}$$

One can further simplify these by using ratio of nuclear volume to cytoplasm volume $\beta =\frac{V_{n}}{V_{c}}$

$$\begin{array}{l} \frac{dn}{dt}=-k_{e}n+k_{i}c\\ \frac{1}{\beta }\frac{dc}{dt}=+k_{e}n-k_{i}c-\eta c \end{array}$$

By substituting *k_i_*, we get following equations to solve ultimately:

(eq. 1) $$\frac{dn}{dt}=-\left(k_{e}\right)n+\left(k_{e}\alpha \right)c$$

(eq. 2) $$\frac{dc}{dt}=+\left(\beta k_{e}\right)n-\left(\beta k_{e}\alpha +\beta \eta \right)c$$

We then solve these equations numerically using MATLAB function ode15s, and fit them to the experimental data to get influx/efflux rates and bleaching rates. Variables in bold are the unknowns to be fitted with fminsearch function in MATLAB (R2020b).

### FLIP Imaging and Analysis

For quantification of FLIP (Fluorescent Loss In Photobleaching) experiments, we followed the fluorescence intensities of three different regions, segmented manually: nucleus, cell, and background. Image acquisition was done with a Zeiss LSM880 inverted confocal microscope objective and using using Zeiss ZEN2.3 SP1 FP3 (black, version 14.0.24.201), using a 63X 1.46 NA oil immersion objective and a 488nm wavelength argon laser. We used a bleaching ROI of 17 × 17 (~12.9 μm^2^) pixels. 10 baseline images were acquired every 3 seconds before photobleaching. Then, every 3 seconds (during a total of 120 seconds) the ROI was photobleached, and an image of 512 × 512 pixels was acquired. The power of the laser used to bleach was adjusted to result in the same bleaching rate η. Due to differences in cell morphology, this corresponded to 60% power for cells on 1.5 kPa substrates, and 100% power for cells on 30 kPa substrates. This difference occurred because cells were more rounded on soft gels and therefore thicker in the z axis, leading to a taller column of cytoplasm affected by photobleaching. Cells with beaching rates above 0.12 were discarded. We note that differences in obtained rates between 1.5/30 kPa substrates were reproduced when comparing cells at 30 kPa with/without DN KASH overexpression, where cell morphologies and bleaching laser power was not altered. In the mathematical model, the transport between nucleus and cytoplasm is modelled as transport between two compartments, where the cytoplasm is continuously bleached. We assume that the concentration of protein is uniform in each compartment and that during steady state (before photobleaching) the ratio (α) between nucleus and cytoplasm’s protein concentration is constant. The ROIs identified for nucleus and cytoplasm were narrow rings around the nucleus, either inside or outside of the nucleus. The average fluorescence intensity of these regions was used as a proxy for nuclear concentration (*n*) and cytoplasmic concentration (*c*). The intensities were corrected for background noise, and normalized by the total integrated cell intensity. Experimental data for n and c was used to solve equations 1 and 2, as explained above. The ratio of concentrations at steady state (α) was taken as n/c at the initial timepoint (before photobleaching). To calculate the ratio of nuclear to cytoplasmic volume (β), we first took confocal stacks of cells with a nuclear fluorescent label (DAPI) and whole cell fluorescent label (GFP), seeded on both 1.5 kPa and 30 kPa gels. In those cells, we noted an excellent correlation between the nuclear/cytoplasmic ratio volume ratio β, and the nuclear/cytosolic area ratio, calculated with nuclear and cytosolic areas at a representative central slice of the cell (Extended Data Fig. 1). Thus, in FLIP experiments we measured area ratios from images, and converted this to volume ratios using the experimental correlation.

To solve for unknown variables, we used a curve fitting technique with a weighted least square method. The experimental data for concentrations (*n*,*c*) is fitted to a solution of the ODEs (*n_f_*, *c_f_*). The objective function *f* is then formulated as the sum of squares of residuals of model and experimental data as:

$$f=\sum_{t}w_{n}{\left(n-n_{f}\right)}^{2}+w_{c}{\left(c-c_{f}\right)}^{2}$$

*Where w_n_* and *W_c_* are used to weigh the function by time and compartment concentration to avoid bias in the fitting:

$$w_{n}=\frac{1}{(t+\epsilon )\sum_{t}n}\;w_{c}=\frac{1}{(t+\epsilon )\sum_{t}c}$$

Here, *w_n_*, *w_c_*, *n*, *c*, and *n_f_*, *c_f_* are all a function of time *t* and *∊* is an arbitrary scalar constant (set to 10) used simply to prevent the denominator of *w_n_* and *w_c_* from reaching zero. We use the fminsearch function of MATLAB to minimize *f* as a function of ODE parameters *k_e_* and *η* (equations 1 and 2). For each iteration, *n_f_*, *c_f_* is calculated as a function of *k_e_* and *η* using the Matlab ode15s solver. We note that resulting fitted rates showed more variability for conditions with fast rates (corresponding to small molecular weight constructs) than conditions with slow rates (see Fig. 2e,f). This is likely caused by a higher experimental error in measuring fast rates: in cells with faster rates, photobleaching occurs faster, and therefore the important part of the fluorescence intensity curves is compressed in a shorter interval (less frames). This makes the subsequent fitting more susceptible to noise.

### Statistical and reproducibility

Statistical analysis was performed with GraphPad Prism 9.0.0. When testing data with a 2-way ANOVA, we transformed the data (y=log10(y)) which showed smaller residuals, and therefore better statistical power, when transformed.

All micrograph images shown in the figures are representative examples of results from 3 different experiments.

## Extended Data

**Extended data figure 8:**
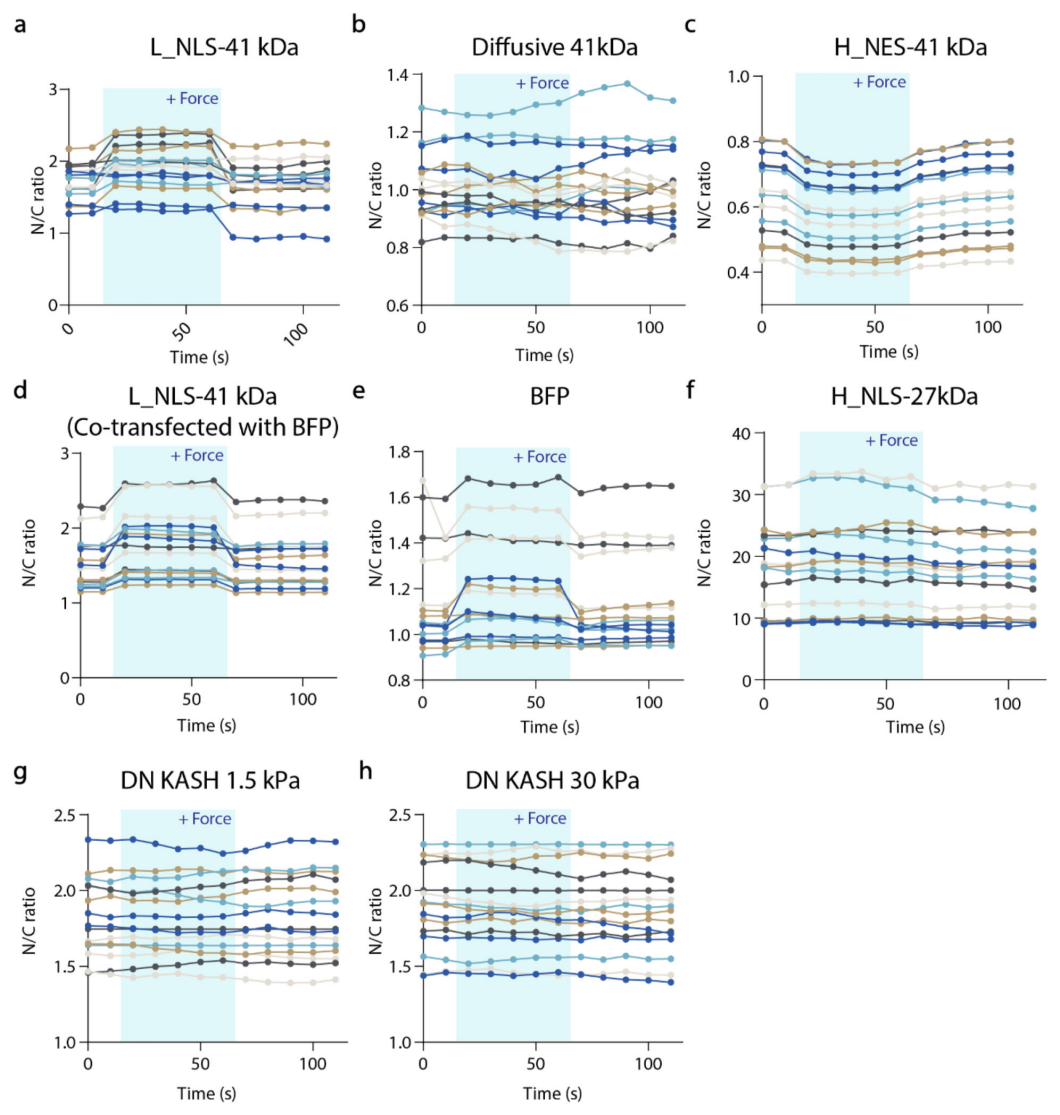
Plots showing the evolution with time of N/C ratios before, during and after force application to the cell nucleus for all cells measured. a-b) AFM experiments reported in Figure 3, c) Figure 5, and d-h) Extended Data Figure 3. Source numerical data are available in source data.

## Supplementary Material

Supplementary Material

## Data Availability

Source data have been provided in Source Data. All other data supporting the findings of this study are available from the corresponding author on reasonable request

## Code availability

All codes generated during the current study are available from the corresponding authors on reasonable request. The kinetic mathematical model of transport is available at https://github.com/ravehlab/npctransport_kinetic.
