# Supplementary material for: Mechanical force application to the nucleus regulates nucleocytoplasmic transport
Source: Nat Cell Biol. Author manuscript; Available in PMC 2023 Jul 19. (PMC7614780; doi:10.1038/s41556-022-00927-7)
Supplement: Supplementary Material [file EMS179322-supplement-Supplementary_Material.pdf]

Supplementary Note

Modelling of mechanosensitive nucleocytoplasmic transport

<u>Initial conceptual model</u>

To obtain a first understanding of how mechanical force should affect nucleocytoplasmic transport of constructs with NLS sequences, we developed a simple conceptual model. For this, we simply assumed that:

$$\frac{n}{c} = \frac{f_i}{f_e} = \frac{f_p + f_f}{f_p} \text{ if } \frac{n}{c} < \left(\frac{n}{c}\right)_{sat}$$

$$\frac{n}{c} = \left(\frac{n}{c}\right)_{sat} \text{ otherwise}$$

Where n/c is the nuclear to cytoplasmic concentration ratio of a given construct, f_i and f_e are the flow rates in and out of the nucleus respectively, f_p is a passive diffusive flow rate through NPCs which decreases with increasing MW (and is equal in the export and import direction), f_f is a facilitated diffusive flow rate which depends on the strength of the NLS sequence (and does not depend on MW) and $\left(\frac{n}{c}\right)_{sat}$ is a maximum value for n/c ratios, where saturation is reached. Such saturation would imply that any change in f_i is matched by an equivalent change in f_e, keeping the ratio constant. In this initial conceptual model, the underlying reason for this is not addressed. However, the detailed, kinetic model described below provides a justification for this, discussed in the text: as NLS affinities increase, cargo molecules can compete with Ran-GTP for binding importins, limiting the ability of Ran-GTP to disassemble the cargo-importin complex. This leads to the facilitated diffusion of importin-cargo complexes out of (and not only into) the nucleus. In this scenario, both influx and efflux are driven by facilitated diffusion, and respond in the same way.

Note that facilitated and passive diffusion are assumed to have additive contributions to total influx flow rates, and that for simplicity changes in nuclear/cytosolic volume compartments are not considered (unlike in the more detailed, kinetic model below). The effect of force applied to the nucleus is introduced by increasing f_p by two-fold at the lowest MW (arbitrarily set to have to have a value of f_p=1 in the absence of force) and by a progressively smaller amount as MW increases, until having a negligible effect at the highest MW (arbitrarily set to have a value of f_p=0.015 in the absence of force). Force also increases f_f by 2-fold, in this case independently of MW. After applying these effects of force, mechanosensitivity is calculated as:

$$mechanosensitivity = \frac{\left(\frac{n}{c}\right)_{stiff}}{\left(\frac{n}{c}\right)_{soft}}$$

Graphs in Fig. 4a,b were calculated by calculating n/c and mechanosensitivity for a range of values of f_p (1-0.015 before force application) and f_f, (16-0.12 before force application). The choice of values is arbitrary, and merely intends to show the relative effects when either f_f or f_p dominate the overall n/c ratio. Accordingly, no specific numerical values are shown in the graphs.

Kinetic mathematical model of transport.

The kinetic model of nucleocytoplasmic transport (Model Figure 1, Model Tables 1-3) was constructed following a canonical description of the nucleocytoplasmic transport process[1–4]. A system of ordinary differential equations (Model Table 1) is used to describe passive diffusion of unbound cargo molecules through NPCs; Ran-mediated facilitated diffusion of cargo:importin complexes through NPCs, and maintenance of the RanGTP gradient across the nuclear envelope through NTF2-mediated import of RanGDP[5,6], RanGAP-mediated hydrolysis of RanGTP to RanGDP in the cytoplasm[7], and chromatin-bound RCC1 (RanGEF) mediated conversion of RanGDP to RanGTP in the nucleus[8]. During passive diffusion, unbound cargo molecules diffuse in either direction at a rate proportional to their concentrations, in accordance with Fick's law[9,10]. During facilitated diffusion, cargo:importin complexes interact with docking sites on NPCs, diffuse across the nuclear envelope and release cargo by interacting with RanGTP. Docking rate to the NPC is proportional to the number of available docking sites. Cargo and importin molecules also associate and dissociate spontaneously in a non-Ran dependent manner. Of note, the model does not consider competition of cargo with other, endogenous cargo molecules already present in cells[11], due to the difficulty in estimating overall endogenous cargo concentrations or affinities. However, the main effect of this competition is to limit the availability of Ran, something which is already considered by modelling a finite Ran concentration.

Model parametrization: The kinetic model of transport provides a simplified minimal description of the transport process based on a set of canonical assumptions[1–4]. It is not meant to reproduce precise empirical values, rather to characterize dependencies among key biophysical parameters that determine NPC transport kinetics on soft and stiff surfaces. Nonetheless, the model has been carefully parametrized to reproduce key features of transport, and it is remarkably robust to changes in its parameter values. Unless stated otherwise, all simulations were conducted using the mean measured nuclear and cytoplasmic volumes of 627 fL and 2194 fL in our dataset. Passive diffusion rates for different cargo molecules of different sizes were also obtained from measurements (Fig. 2e,f). The cargo concentration was estimated to be in the range 0.01-0.1 μM, based on comparison of GFP fluorescence values and reference fluorescence of purified GFP. This is much lower than the ~10 μM physiological concentrations of importins such as Kapß1[12,13], and the estimated 5-20 μM concentration of RanGTP concentration in HeLa cells[4], thus precise values of these parameters are expected to have limited effect. Indeed, doubling or halving Ran concentration had limited qualitative effect on our model results. The Ran cycle kinetic parameters were fitted to reproduce a robust nuclear:cytoplasmic RanGTP ratio of >500[4], starting from a 1000:1 ratio. The number of dock sites per NPC was estimated from the thousands of FG binding sites per NPC and the large fraction of cargo and NTR molecules found in mass-spectrometry measurements in native NPCs[14].

Simulation code. Our simulations were implemented in Python (version 3.6). They are fully reproducible; the source code and the run parameters can be found in https://github.com/ravehlab/npctransport_kinetic (run03 was used to produce model results in this manuscript).

Model Table 1. Ordinary differential equations (ODEs) of a kinetic model of transport. Subscripts N and C indicate nuclear and cytoplasmic localization. Subscript NPC indicates localization to the NPC, and subscripts NPC-C and NPC-N indicate sub-localization at the nuclear and cytoplasmic sides of the NPC, respectively. Bracketed variables are in units of concentration (for either the nucleus or the cytoplasm) and non-bracketed variables indicate actual numbers of molecules (for NPC-docked <u>molecules</u>) (Table S1). N_A is Avogadro's number.

**ODEs**	**Processes described**
$[\dot{C}_N] = -\pi_{passive}[C_C] \cdot \frac{V_C}{V_N}$<br>$[\dot{C}_C] = -\pi_{passive}[C_N] \cdot \frac{V_N}{V_C}$	Passive diffusion of unbound cargo through the NPC
$\dot{CI}_{NPC-N} = \sigma_{on} \cdot (N_{dock} - CI_{NPC}) \cdot [CI_N] - \sigma_{off} \cdot CI_{NPC-N} + \varphi \cdot (CI_{NPC-C} - CI_{NPC-N}) - \alpha[GTP_N]CI_{NPC-N}$<br><br>$\dot{CI}_{NPC-C} = \sigma_{on} \cdot (N_{dock} - CI_{NPC}) \cdot [CI_C] - \sigma_{off} \cdot CI_{NPC-C} + \varphi \cdot (CI_{NPC-N} - CI_{NPC-C})$<br>$[\dot{C}_N] = [GTP_N] \cdot \left(\frac{\alpha CI_{NPC-N}}{N_A \cdot V_N} + \beta[CI_N]\right) + k_{off}[CI_N]$<br>$[\dot{CI}_N] = -\beta[GTP_N][CI_N] - k_{off}[CI_N] - \sigma_{on} \cdot (N_{dock} - CI_{NPC}) \cdot [CI_N]/(N_A \cdot V_N) + \sigma_{off} \cdot CI_{NPC-N}/(N_A \cdot V_N)$	Facilitated diffusion:<br>- Docking and undocking of cargo:importin complexes to and from NPCs, resp.<br>- NPC traversal of NPC-docked cargo:importin complexes between cytoplasmic and nuclear ends of the NPC<br>- RanGTP-dependent and RanGTP-independent dissociation of cargo:importin complexes in the nucleus and NPC
$[\dot{C}_C] = k_{off}[CI_C]$<br>$[\dot{CI}_C] = -k_{off}[CI_C] - \sigma \cdot (N_{dock} - CI_{NPC}) \cdot [CI_C]/(N_A \cdot V_C) + \sigma_{off} \cdot CI_{NPC-C}/(N_A \cdot V_C)$	Non-RanGTP dependent dissociation of cargo molecules from importin molecules in the cytoplasm
$[\dot{CI}_N] = k_{on}[C_N]$<br>$[\dot{CI}_C] = k_{on}[C_C]$<br>$[\dot{C}_N] = -k_{on}[C_N]$<br>$[\dot{C}_C] = -k_{on}[C_C]$	Association of cargo molecules to importin molecules. assuming [I]>>[C] (see Model parametrization)
$[G\dot{T}P_N] = \gamma[GDP_N] - (\delta + \epsilon)[GTP_N] - [GTP_N] \cdot \left(\frac{\alpha CI_{NPC-N}}{N_A \cdot V_N} + \beta[CI_N]\right)$<br>$[G\dot{T}P_C] = \epsilon[GTP_N] \cdot \frac{V_N}{V_C} - \eta[GDP_C] + [GTP_N] \cdot \left(\frac{\alpha CI_{NPC-N}}{N_A \cdot V_C} + \beta[CI_N] \cdot \frac{V_N}{V_C}\right)$<br>$[G\dot{D}P_N] = \delta[GTP_N] + \zeta[GDP_C] \cdot \frac{V_C}{V_N}$<br>$[G\dot{D}P_C] = \eta[GTP_C] - \zeta[GDP_N] \cdot \frac{V_N}{V_C}$<br>$[G\dot{D}P_C] = -\zeta[GDP_N] \cdot \frac{V_N}{V_C}$	Ran cycle:<br>- RCC1 (RanGEF) mediated exchange of RanGDP to RanGTP<br>- RanGAP-mediated hydrolysis of RanGTP to RanGDP<br>- Residual reverse conversion of nuclear RanGTP to RanGDP<br>- NTF2-mediated transport of RanGDP (symmetric for export and import, results in net import due to concentration gradient)<br>- export of importin-bound RanGTP following an import cycle (the exported importins are not modeled explicitly)

Model Table 2. Kinetic model variables.

Variable name	Description
C	cargo molecules (unbound)
CI	cargo:importin complex (bound)
GTP	RanGTP
GDP	RanGDP

Model Table 3. ODE model coefficients.

Model coefficient	Description	Value*	units
α	Rate of GTP-dependent conversion of NPC-docked cargo:importin complex to nuclear cargo	10^6	$M^{-1}sec^{-1}$
β	Rate of GTP-dependent conversion of nuclear cargo:importin complex to nuclear cargo	10^6	$M^{-1}sec^{-1}$
γ	Rate of exchange of GDP_N to GTP_N by RCC1	1000	sec^{-1}
δ	Rate of residual exchange of GTP_N to GDP_N by RCC1	0.2	sec^{-1}
ϵ	Rate of RanGTP passive export	0.5	sec^{-1}
ζ	Rate of NTF2-mediated RanGDP transport	1.0	sec^{-1}
η	Rate of RanGAP-mediated hydrolysis of RanGTP to RanGDP	500.0	sec^{-1}
k_{on}	Rate of cargo association to importin molecules	0.001-3.83**	sec^{-1}
k_{off}	Rate of dissociation of cargo:importin complexes	0.05	sec^{-1}
$\pi_{passive}$	Passive diffusion rate (permeability)	0.03-0.16***	sec^{-1}
σ_{on}	Rate of docking of cargo:importin complexes to NPC	50×10^6	$sec^{-1}M^{-1}$
σ_{off}	Rate of undocking of cargo:importin complexes from NPC	3000.0	sec^{-1}
φ	Traversal rate of cargo:importin complexes across the NPC	15.0 (soft) 150.0 (stiff)	sec^{-1}
N_{dock}	Number of docking sites on NPCs	500	-
N_{NPC}	Number of NPC molecules per cell	2000	-
$[Ran]_{cell}$	Total concentration of RanGTP and RanGDP in the entire cell	20	μM
$[C]_{cyto,t=0}$	Initial cytoplasmic concentration of cargo molecules****	0.1	μM
V_N	Nuclear volumen	627×10^{-15}	L
V_C	Cytoplasmic volume	2194×10^{-15}	L
Δt	Simulation timestep	0.001	sec
τ	Simulation time	100	sec

* unless stated otherwise for specific runs

** 0.054 sec^{-1} for weak NLS, 0.205 sec^{-1} for medium NLS

*** according to measurements of actual passive diffusion rates for different cargo molecules (Fig. 2e,f)

**** the initial nuclear concentration is zero in all runs

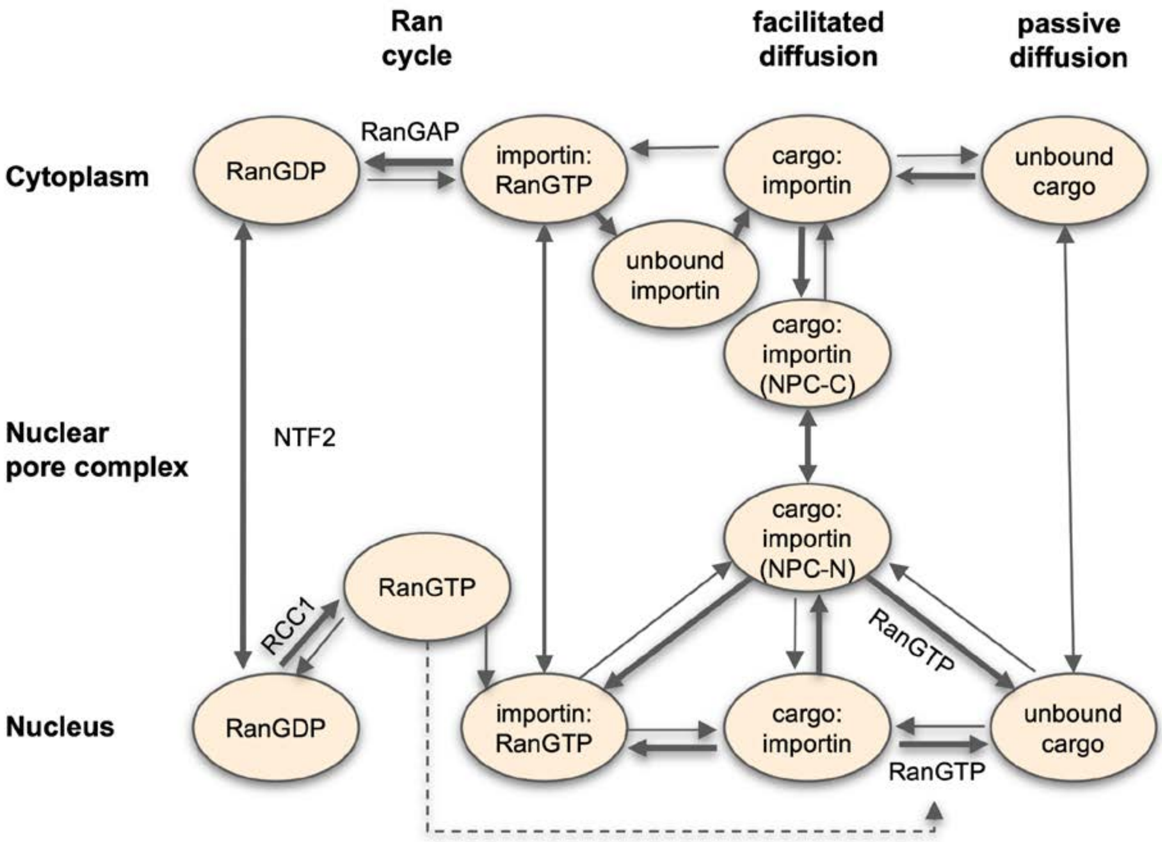

Model Figure 1. Kinetic model of import through the NPC. The concentration of importin molecules is not modeled explicitly (see Text), except to indicate whether cargo molecules are in the bound or unbound state, but they are shown here for completeness.

References

1. Kim, S. & Elbaum, M. A Simple Kinetic Model with Explicit Predictions for Nuclear Transport. *Biophysical Journal* **105**, 565–569 (2013).

2. Cautain, B., Hill, R., De Pedro, N. & Link, W. Components and regulation of nuclear transport processes. *FEBS Journal* vol. 282 445–462 (2015).

3. Jovanovic-Talisman, T. & Zilman, A. Protein Transport by the Nuclear Pore Complex: Simple Biophysics of a Complex Biomachine. *Biophysical Journal* vol. 113 6–14 (2017).

4. Görlich, D., Seewald, M. J. & Ribbeck, K. Characterization of Ran-driven cargo transport and the RanGTPase system by kinetic measurements and computer simulation. *The EMBO Journal* **22**, 1088–1100 (2003).

5. A, S., A, B. & IG, M. Nuclear import of Ran is mediated by the transport factor NTF2. *Current biology : CB* **8**, 1403–1406 (1998).

6. Ribbeck, K., Lipowsky, G., Kent, H. M., Stewart, M. & Görlich, D. NTF2 mediates nuclear import of Ran. *The EMBO Journal* **17**, 6587–6598 (1998).

7. Bischoff, F. R., Klebe, C., Kretschmer, J., Wittinghofer, A. & Ponstingl, H. RanGAP1 induces GTPase activity of nuclear Ras-related Ran. *Proc Natl Acad Sci U S A* **91**, 2587–2591 (1994).

8. L, R., J, K., A, H. & A, W. Structural basis for guanine nucleotide exchange on Ran by the regulator of chromosome condensation (RCC1). *Cell* **105**, 245–255 (2001).

9. Timney, B. L. *et al.* Simple rules for passive diffusion through the nuclear pore complex. *Journal of Cell Biology* **215**, 57–76 (2016).

10. Ribbeck, K. & Görlich, D. Kinetic analysis of translocation through nuclear pore complexes. *The EMBO Journal* **20**, 1320 (2001).

11. Kopito, R. B. & Elbaum, M. Nucleocytoplasmic transport: A thermodynamic mechanism. *http://dx.doi.org/10.2976/1.3080807* **3**, 130–141 (2010).

12. Kapinos, L. E., Huang, B., Rencurel, C. & Lim, R. Y. H. Karyopherins regulate nuclear pore complex barrier and transport function. *Journal of Cell Biology* **216**, 3609–3624 (2017).

13. Paradise, A., Levin, M. K., Korza, G. & Carson, J. H. Significant Proportions of Nuclear Transport Proteins with Reduced Intracellular Mobilities Resolved by Fluorescence Correlation Spectroscopy. *Journal of Molecular Biology* **365**, 50–65 (2007).

14. Kim, S. J. *et al.* Integrative structure and functional anatomy of a nuclear pore complex. *Nature 2018 555:7697* **555**, 475–482 (2018).
